# BMI trajectories after primary school-based lifestyle intervention: Unravelling an uncertain future. A mixed methods study

**DOI:** 10.1016/j.pmedr.2021.101314

**Published:** 2021-01-07

**Authors:** Marije Oosterhoff, Shahab Jolani, Daisy De Bruijn-Geraets, Anoukh van Giessen, Hans Bosma, Onno C.P. van Schayck, Manuela A. Joore

**Affiliations:** a Department of Clinical Epidemiology and Medical Technology Assessment (KEMTA) Maastricht University Medical Centre MUMC+, Care and Public Health Research Institute (CAPHRI), Maastricht University, Maastricht, the Netherlands; b Department of Methodology and Statistics, Care and Public Health Research Institute (CAPHRI), Maastricht University, Maastricht, the Netherlands; c Center for Prevention and Health Services, National Institute of Public Health and the Environment, Bilthoven, the Netherlands; d Department of Social Medicine, Care and Public Health Research Institute (CAPHRI), Maastricht University, Maastricht, the Netherlands; e Department of Family Medicine, Care and Public Health Research Institute (CAPHRI), Maastricht University, Maastricht, the Netherlands

**Keywords:** BMI, body mass index, HPSF, the Healthy Primary School of the Future, PAS, the physical activity school, SES, socioeconomic status, Body mass index, Trajectory, Child, Health promotion/economics*

## Abstract

This mixed methods study aimed to examine plausible body mass index (BMI) trajectories after exposure to a primary school-based lifestyle intervention to aid in estimating the long-term intervention benefits.

BMI trajectories for children at control schools (mean 7.6 years of age) were modelled until 20 years of age through extrapolating trial evidence (N = 1647). A reference scenario assumed that the observed 2-year effects of the ‘Healthy Primary Schools of the Future’ (HPSF) and ‘Physical Activity Schools’ (PAS) were fully maintained over time. This was modelled by applying the observed 2-year BMI effects until 20 years of age.

Expert opinions on likely trends in effect maintenance after the 2-year intervention period were elicited qualitatively and quantitatively, and were used for developing alternative scenarios.

Expert elicitation revealed three scenarios: **(a)** a constant exposure-effect and an uncontrolled environment with effect decay scenario, **(b)** a household multiplier and an uncontrolled environment with effect decay scenario, and **(c)** a household multiplier and maintainer scenario. The relative effect of HPSF at 20 years of age was −0.21 kg/m^2^ under the reference scenario, and varied from −0.04 kg/m^2^**(a)** to −0.06 kg/m^2^**(b)**, and −0.50 kg/m^2^**(c)**. For PAS, the relative effect was −0.17 kg/m^2^ under the reference scenario, and varied from −0.04 kg/m^2^**(a, b)**, to −0.21 kg/m^2^**(c)**.

The mixed methods approach proved to be useful in modelling plausible BMI trajectories and specifying uncertainty on effect maintenance. Further observations until adulthood could reduce the uncertainty around future benefits. This trial was retrospectively registered at Clinicaltrials.gov (NCT02800616).

## Highlights

• We modelled body mass index development after school-based lifestyle intervention.
• We combined empirical data and expert elicitation.
• Expert elicitation revealed three scenarios with varying effect maintenance.
• The mixed methods approach proved useful in specifying uncertainty.
• Results could be used to communicate uncertainty to decision-makers.

## 1 Background

Primary school-based lifestyle interventions aim to optimize children’s health and wellbeing by providing opportunities for changing lifestyle behaviours like physical activity and nutrition behaviours. While the investments for intervention implementation have to be made early in life, the main health benefits and cost savings may only be realized beyond the timeframe of empirical studies and beyond childhood (Hamilton et al., 2018, Jones et al., 2011).

Population health models are available, which link short-term intervention impacts to chronic disease risk. Body mass index (BMI) is frequently used as a risk factor in population health models for the evaluation of school interventions targeting physical activity and/or diet (Oosterhoff et al., 2018). Available population health models for the Dutch population, however, start in adulthood (Hoogenveen et al., 2010). To estimate the long-term impacts of primary school-based lifestyle interventions, the gap between childhood (observation range of empirical studies) and adulthood (captured in population health models) has to be filled. Few empirical studies included long-term follow-up measurements after primary school-based lifestyle interventions in order to assess the effect maintenance (James et al., 2007). Whilst some studies found a fast decay of the intervention effect (Oosterhoff et al., 2018, Hoogenveen et al., 2010), others reported some sustained effects in the overall study sample or in subgroups only (e.g. children with a high socioeconomic background) (Marcus et al., 2000, Taylor et al., 2008). In modelling long-term benefits, several cost-effectiveness studies on primary-school based lifestyle interventions have used a range of alternative ‘hypothetical’ estimates on effect maintenance (Rush et al., 2014, Ekwaru et al., 2017, Ananthapavan et al., 2020, Brown et al., 2019, Dalziel and Segal, 2006). These results show that estimates on the maintenance of the intervention effect are paramount in modelling the long-term benefits of childhood lifestyle interventions (Ananthapavan et al., 2020, Brown et al., 2019, Dalziel and Segal, 2006). This also indicates that making transparent and plausible assumptions on the effect maintenance is crucial to adequately inform implementation and funding decisions on primary school-based lifestyle interventions.

In a quasi-experimental study in the Southern region of the Netherlands, two interventions were evaluated. The ‘Healthy Primary School of the Future’ (HPSF) implemented a daily healthy lunch and daily physical activity sessions, whereas the ‘Physical Activity School’ (PAS) focused on physical activity only (Willeboordse et al., 2016). After one and two years it was found that standardized BMI scores decreased in children from HPSF and PAS as compared to children from control schools (Willeboordse et al., 2016). The objectives of the current study are to: 1) model a BMI trajectory for children from control schools from primary school up to young adulthood, and 2) model the BMI trajectories for children from HPSF and PAS, including plausible intervention effect maintenance over time.

## 2 Methods

### 2.1 The ‘healthy Primary School Of The Future’ initiative

The ‘Healthy Primary School of the Future’ (HPSF) initiative aims to integrate health promotion in the primary school setting. In the Netherlands, children attend primary school for eight years (grade 1–8, from 4 to 12 years of age). Central to the HPSF initiative are the provision of a daily healthy lunch and daily structured physical activity sessions, which is uncommon in Dutch primary schools. Two schools decided to implement both changes and are referred to as the ‘Healthy Primary School of the Future’ (HPSF). Two other schools decided to implement the daily structured physical activity program only and are named the ‘Physical Activity School’ (PAS) (Willeboordse et al., 2016). In a quasi-experimental study, both interventions were examined and compared to schools that maintained the regular curriculum (control schools). Details on the quasi-experimental study and a process evaluation have been previously published (Willeboordse et al., 2016, Bartelink et al., 2019a, Bartelink et al., 2019b).

### 2.2 Study sample and growth data

All children from study year one to eight (age 4 to 12) enrolled at the eight participating schools were eligible to participate in the study, which is internationally comparable to two years of Kindergarten and six grades. The study sample was influenced by the dynamic character of a school population (new children enter school each year, while others finish school). The study sample of the present study consists of all children who had a two-year intervention exposure by including all children who were enrolled at the schools from baseline onwards (with or without participation in the measurements at baseline) (Bartelink et al., 2019a). Of the remaining N = 1676, a total of N = 1647 were included in the data analysis as complete data on sex, age, and socioeconomic status (SES) and BMI data on at least one time point were available (see flow diagram in Additional file 1). During annual measurements, trained research assistants measured children’s height and weight (measured to the nearest 0.1 cm and 0.1 kg) (September-November from school year 2015/2016 to 2019/2020). Children were measured with light clothing and no shoes. Information on children’s age (in years) and sex was retrieved from the educational board. BMI values were calculated from age 5 to 12 years, because height and weight was measured from age 5 onwards, and because only few children in the last grade did already turn 12 years at the start of the school year. SES was based on information from a parent-reported questionnaire, and calculated as the mean of standard scores on maternal education, paternal education, household income (adjusted for household size), and neighbourhood SES score (latter derived from the Netherlands Institute for Social Research). SES was categorized (low, average, high) based on tertile scores.

### 2.3 Unobserved effects: Two phases

We distinguished two phases: 1) primary school period, and 2) after the primary school period up to 20 years.

### 2.4 Estimation of BMI trajectories without expert information

#### 2.4.1 BMI trajectory of children at control schools

##### 2.4.1.1 Phase 1: Primary school period

To model the BMI growth trajectory at a group-level (instead of accurately resembling individual BMI development) we modelled average BMI-trajectories based on the raw BMI scale. Two models were compared: a linear mixed model and a piecewise mixed model as both have been used in the literature (Box 1). BMI values were log-transformed to accommodate for the right-skew of BMI-values for older children (Buscot et al., 2017, Graversen et al., 2017). Both mixed models used all available data (with BMI data on at least one time point) under the missing at random assumption as BMI data was unavailable for 1.7% (N = 28) of the original study sample (N = 1676).

Firstly, a linear mixed model was built. Model selection criteria (Akaike information criterion, Bayesian information criterion, Loglikelihood) were used to test whether addition of second and third order effects of age improved the model fit (Box 1). Secondly, a piecewise mixed model (broken stick model) was used to mimic the differences in BMI growth at different ages (Buscot et al., 2017). A breakpoint at age 6 was used to reflect the transition in children’s BMI growth rates between 4 and 6 years of age (Tilling et al., 2014). Additionally, a breakpoint at the age of nine years was selected to reflect the timing of the mid-growth spurt that may occur at pre-puberty (Robinson et al., 2019). The two models included random intercepts and a random slope (linear effect of age) in order to model individual BMI development and account for the correlation of repeated measurements within children (unstructured covariance structure) (Robinson et al., 2019). Models were fitted for boys and girls separately. The models were adjusted for SES due to the SES-specific BMI trends in the study sample (Bartelink et al., 2019a). Dummy variables for HPSF and PAS (and HPSF/PAS * age) were added in the fixed part of both models to control for the baseline differences between the three school types.

Next, BMI values were estimated for children at control schools with an average SES background. Results were stratified for each age- and sex strata (in years). This was done in order to specify the BMI trajectory at the group-level and to be able to deduct the overall relative effects of HPSF and PAS in the next steps. All analyses were performed in R version 3.5.1.

Box 1 **. BMI trajectory models.**

Model 1: Linear mixed model *

BMI_i,t_ = β_0,i_ + β_1,i_*Age_i,t_ + β_2_*Age_i,t_^^2^ + β_3_*Age_i,t_^^3^ + β_4_*HPSF_i_ + β_5_*PAS_i_ +.

β_6_ (HPSF_i_* Age_i,t_) + β_7_ (PAS_i_* Age_i,t_) + β_8_*lowSES_i_ + β_9_*highSES_i_ + e_i,t._

**Notes:** BMI_i,t_ = BMI value at a specific age of the child, t = time point (baseline, year 1, year 2), i = individual (N = 1676), βo,i = random intercept parameter, Age_i,t_ = age (in years) of the child, β_1,i_ = random linear slope parameter, β_2,i_ = quadratic slope parameter, β_3,i_ = cubic slope parameter, HPSF_i,t_ = Healthy Primary School of the Future, PAS_i,t_ = Physical Activity School, lowSES_i,t_ = low socioeconomic background, lowSES_i,t_ = high socioeconomic background, e_i,t_ = error term (residual variance).

*Addition of the second order and third order effects for age was assessed by comparing the the Loglikelihood, Akaike information criterion (AIC), and the Bayesian information criterion to the null model (only first order effects of age). The Loglikelihood and AIC showed that a model predicting lnBMI by the third order effects of age showed a good fit to our data, and this model has previously been used to model BMI trajectories in European youth (14).

Model 2: Piecewise mixed model.

BMI_i,t_ = β_0,i_ + β_1,i_*Age_i,t_ + β_2,i_*D(Age, ≥6 years) + β_3,i_*D(Age, ≥9 years) + β_4_*HPSF_i_ + β_5_*PAS_i_ + β_6_(HPSF_i_*D[Age, ≥6 years]_i_) + β_7_(PAS_i_*D[Age, ≥6 years]_i_) + β_8_(HPSF_i_*D[Age, ≥9 years]_i_) + β_9_(PAS_i_*D[Age, ≥9 years]_i_) + β_10_*lowSES_i_ + β_11_*highSES_i_ e_i,t._

**Notes:** BMI_i,t_ = BMI value at a specific age of the child, t = time point (baseline, year 1, year 2), i = individual (N = 1676)**,** βo,i = random intercept parameter, Age_i,t_ = age (in years) of the child, D(Age, ≥6 years) = dummy variable (1=≥6 years, 0=<6 years), D(Age, ≥9 years) = dummy variable (1=≥9 years, 0=<9 years), HPSF_i,t_ = Healthy Primary School of the Future, PAS_i,t_ = Physical Activity School, lowSES_i,t_ = low socioeconomic background, lowSES_i,t_ = high socioeconomic background, e_i,t_ = error term (residual variance).

##### 2.4.1.2 Phase 2: After the primary school period

BMI trajectories for the control group were extrapolated to the period after primary school (12 up to 20 years of age) by using the modelled parameter estimates for obtaining BMI values at age 12–20 years. To examine whether the extrapolated models yielded plausible BMI trajectories, the face validity of the fitted results was inspected. Crossectional data from the Fifth Dutch Growth Study, representing BMI values among Dutch youth in 2009, was used for inspecting face validity (Schönbeck et al., 2011).

### 2.5 BMI trajectory of children at HPSF and PAS (reference scenario)

In a reference scenario, the trajectories for children at ‘Healthy Primary Schools of the Future’ (HPSF) and ‘Physical Activity Schools’ (PAS) were modelled by lowering the BMI trajectory of children at control schools (see above). The BMI trajectory was lowered by deducting the BMI reductions (HPSF and PAS vs. control schools) that were obtained after a 2-year intervention period. The BMI trajectories were lowered from age 6 (2-year effects settling in from age six onwards [2yearsafterthestartofprimaryschoolandinterventionexposure]) until 20 years of age. The reference scenario assumes that the observed 2-year effect are maintained until young adulthood. In the next section, the reference scenario is compared to other effect maintenance scenarios as specified by experts. The effects on children’s standardized BMI values (BMI z-score) after two-years of intervention were previously analysed (Bartelink et al., 2019a). In line with the literature on BMI in children, the effects of HPSF and PAS have been analysed by means of BMI z-scores. To the best of our knowledge, no guidelines exist with regards to the methods for converting the intervention effects on BMI-z to BMI. We used the standard deviation of BMI of the study sample at baseline (SD = 2.55), which was seen as appropriate by the interviewed experts. The overall 2-year intervention effects were applied, as no statistically significant differences between age groups, sex, and SES groups were found (Bartelink et al., 2019a). Adapting the whole trajectory (reference scenario) based on short-term (observed) effects is in accordance with the approach of previous cost-effectiveness studies (Oosterhoff et al., 2018).

### 2.6 Estimation of BMI trajectories with expert information

To obtain a representative and information-rich panel, experts were selected by purposive sampling (N = 11) (Zondervan-Zwijnenburg et al., 2017). A semi-structured interview was performed face-to-face or by telephone (interview led by MO), and followed a pre-developed interview guide consisting of qualitative and quantitative questions (see Additional file 2 for more details). We aimed to elicit experts’ views on the future trends in effect maintenance and the underlying mechanisms through qualitative questions, as well as eliciting quantitative estimates on the future relative effects and the corresponding uncertainty. For the quantitative part, graphical displays from the interactive MATCH Uncertainty Elicitation Tool were used (Roulette method) to specify the mean relative effect/effect maintenance and the variance (probability distribution) during and after the primary school period (Morris et al., 2014).

The qualitative answers were first analysed. Transcriptions were anonymized, and themes relating to the direction of the effect maintenance were identified and grouped in scenarios (thematic content analysis) (MO and DDBG). Scenarios were drafted for the effect maintenance during the primary school period (1) and after the primary school period (2). Scenarios were summarized by using the context-mechanism-outcome configuration of the realist evaluation method to provide insight in the mechanisms underlying effect maintenance (Pawson and Realist, 2004). A summary of the expert’s answers and the constructed scenarios were given to the experts for feedback (member check), which was incorporated in the revision of the scenarios. Identification of scenarios and data saturation were discussed between MO and DDBG.

After drafting the scenarios, we scored the chosen scenario for each expert and sorted the (individually) elicited probability distributions accordingly. Scenario-specific probability distributions were calculated by averaging the means and variances of the expert probability distributions with equal weights for experts.

Last, scenarios for the primary and post-primary school period were combined based on the combinations that were mentioned by the experts. To reflect the BMI reduction during the post-primary school period, the elicited maintenance factor (in percentage) was multiplied with the elicited effect during the primary school period.

## 3 Results

Characteristics of the study sample (N = 1647) are described in Table 1. The study sample consisted of children (47.6% boys) who were on average 7.6 years old at baseline (standard deviation: 2.2 years). Children at control schools had higher BMI values as compared to their peers at HPSF or PAS (Table 1).

**Table 1:** Characteristics of the study sample at baseline (T0).

		Total (N = 1647)			HPSF (N = 525)	PAS (N = 473)	Control (N = 649)
		*N*	*Missing (%)*π	*%/Mean (±SD)*	*%/Mean (±SD)*	*%/Mean (±SD)*	*%/Mean (±SD)*
Sex (% boys)		1647	0 (0%)	47.6%	47.8%	47.8%	47.3%
Age (years)		1647	0 (0%)	7·6 (±2·16)	7·6 (±2·15)	7·4 (±2·21)	7·6 (±2·12)
Ethnicity (% Western)¥		1008	639 (38.8%)	94.1%	93.1%	96.0%	93.4%
SES (%)Ф	*Lowest tertile*	1647	0 (0%)	31.3%	31.8%	15.9%	42.2%
	*Middle tertile*	··		34.1%	28.8%	42.9%	32.1%
	*Highest tertile*	··		34.5%	39.4%	41.2%	25.7%
BMI z-score §		1109	538 (32.7%)	0·135 (±1·02)	0·051 (±1·01)	0·092 (±0·95)	0·232(±1·07)
BMI	Total	1109	538 (32.7%)	17·04 (±2·55)	16·80 (±2·39)	16·85 (±2·31)	17.37 (±2.81)
	Age 5–7	··		16.15 (±1.78)	16·23 (±1·85)	16·15 (±1·58)	16·10 (±1·90)
	Age 8–10	··		17.41 (±2.67)	16·84 (±2·32)	17·02 (±2·26)	18·08 (±3·00)
	Age 11–13	··		18.28 (±3.10)	18·32 (±3·27)	18·27 (±3·20)	18·27 (±2·92)
Overweight & obesity ^*§^	Total	1109	538 (32.7%)	19.9%	16.5%	17.9%	24.1%
Overweight *		··		15.9%	13.1%	16.2%	17.7%
Obesity *		··		4.0%	3.4%	1.7%	6.4%

Notes: BMI = body mass index, HPSF = Healthy Primary School of the Future, PAS = Physical Activity School, SD = standard deviation, SES = socioeconomic status.

¥ Information on children’s ethnicity was collected from annual parental questionnaires. Ethnicity, being native background, Western background or a Non-Western background was based on the country of birth of both parents. Ethnicity was subsequently divided into Western (including native background) and non-Western background (Bartelink et al., 2019a).

Ф Socioeconomic status (SES) was based on information from a parent-reported questionnaire, and calculated as the mean of standard scores on maternal education, paternal education, household income (adjusted for household size), and neighbourhood SES score (latter derived from the Netherlands Institute for Social Research).

§ Pearson chi-square and ANOVA tests indicated statistically significant differences between the groups (BMI-z: p = 0.034, weight status: p = 0.006), see Bartelink et al. (2019a).

* International Obesity Task Force (IOTF) cut-off values for childhood overweight and obesity.

π Missing data at baseline were due to later participation in the study, incomplete information on ethnicity and SES which were obtained from a parental questionnaire, no measurement of height/weight in the first grade.

### 3.1 Estimation of BMI trajectories without expert information

#### 3.1.1 Phase 1: Primary school period

**Fig. 1:**
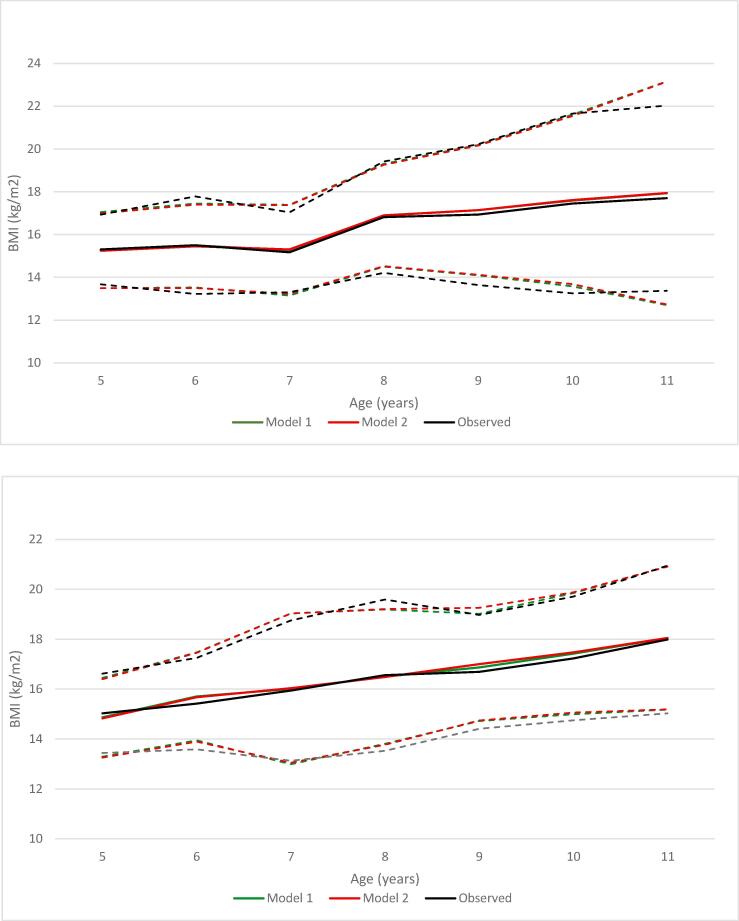
Notes: BMI = body mass index. Solid black line: observed median BMI values. Solid green line: predicted median BMI values based on Model 1 (linear mixed model, see Box 1) Solid red line: predicted median BMI values based on Model 2 (piecewise mixed model, see Box 1) Dashed lines: median – interquartile range; median + interquartile range. * Where separate lines are not visible, the values of Model 1 and Model 2 do overlap. (For interpretation of the references to colour in this figure legend, the reader is referred to the web version of this article.)

The fitted BMI trajectories for boys and girls with an average SES at control schools are presented in Fig. 1. Fitted values showed an increase in variation with age similar to the observed values (see Additional file 3). The trajectory of the observed median BMI values showed a decline for boys at age 7 years, which was also present in the fitted models (Fig. 1, Panel 1).

#### 3.1.2 Phase 2: After the primary school period

Based on the comparison with the Fifth Dutch Growth Study, the decreasing BMI values for boys from 15 years of age in model 1 were not considered plausible, and Model 2 was selected (Additional file 4). For girls, Model 1 adequately reflected the cubic BMI trend. The selection of model 2 for boys and model 1 for girls is supported by literature, which shows that BMI trajectories for boys are relatively linear as compared to a more concave growth in girls due to the early onset of puberty (Cole et al., 2000).

**Fig. 2:**
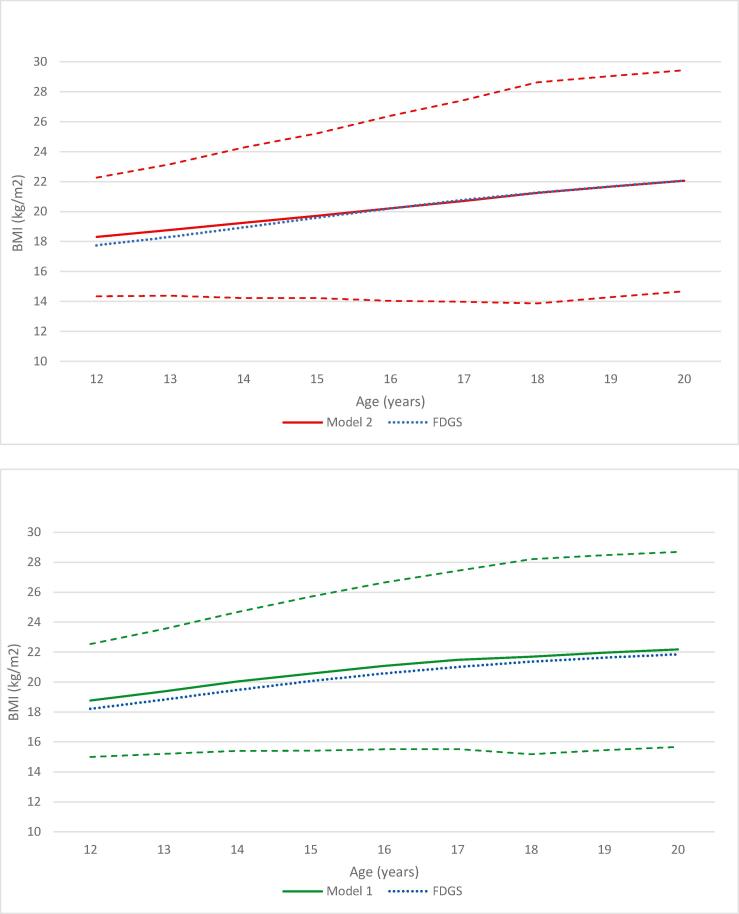
Title: Projected median BMI values for children at control schools at age 12–20 years [average SES]. Panel 1: Boys – model 2 (piecewise mixed model, see Box 1). Panel 2: Girls – model 1 (linear mixed model, see Box 1). Notes: BMI = body mass index, FDGS = Fifth Dutch Growth Study. Solid red line: median projected BMI values. Dashed red lines: median – interquartile range; median + interquartile range. Dashed blue line: median BMI values as observed in the Fifth Dutch Growth Study (Schönbeck et al. (2011)). (For interpretation of the references to colour in this figure legend, the reader is referred to the web version of this article.)

Because the downward slope in BMI growth was not captured from 18 years onwards, the BMI increases from 18 to 19 years and 19–20 years of age in the Fifth Dutch Growth Study were applied. Median BMI values increased to 22.06 (boys) and 22.18 (girls) at age 20 years of age (Fig. 2). In the reference scenario 2-year observed relative effects of HPSF and PAS were applied to the entire trajectory, which resulted in a 0.22 and 0.17 kg/m^2^ lower BMI trajectory, respectively (see details in Additional file 5).

### 3.2 Estimation of BMI trajectories with expert information

#### 3.2.1 Scenarios

##### 3.2.1.1 Phase 1: Primary school period

Expert’s motivations revealed three scenarios (see the description in Table 2 and Additional file 6):

**Table 2:** Expert scenarios regarding the unobserved effects during the primary school period.

Period: Primary school period, 4–12 years of age	
Uncertainty: uncertainty on the (unobserved) relative effects while exposed	
*See Appendix 6 for a selection of relevant literature.*	
Scenario	Description
1. Constant exposure- effect	*Context:* intervention exposure (components, intensity, duration) *Mechanisms:* adaptation to a ‘social norm’ (habituation), weight reductions reach a plateau level after about 2 years. *Outcome:* constant relative effects Description: The evidence base on the maintenance of intervention effects is (relatively) poor. It is most likely that the intervention effects are expected to stay about the same during the entire primary school-period if exposure is prolonged. First of all, children adopt a new ‘social norm’ more easily as compared to adults. After a 2-year period, children are probably used to the adopted behaviour changes. When exposure to the new ‘social norm’ is continued, it is likely that the effects will stay about the same. Body weight changes resulting from reductions in the energy imbalance (e.g. reduced energy intake or increased energy expenditure) generally reach a ‘plateau’ level after about 2 years. In addition to the school environment, health behaviours of parents and peers are important determinants of children’s health behaviours (environment). If family practices stay the same, and if most of the closest peers come from the same schools, it is likely that the relative effects (intervention vs. control) remain stable. “Children are impressionable, mainly through behaviour of parents and peers. When peers are also enrolled at intervention schools, effects will stay about the same” “Effect maintenance should occur within the two-year period”
2. Household multiplier	*Context:* environment (behaviours, facilities, knowledge, norms and beliefs in the household, schools, community, neighbourhood, influence of peers), intervention exposure (components, intensity, duration) *Mechanisms:* effect transfer from the school setting to the household *Outcome:* increased relative effects Description: Continued intervention exposure may not only lead to effect maintenance, but may also induce health behaviour, predominantly via transfer of behaviours to the household setting. Spillover effects may occur, as obesity and related lifestyle behaviours spread through social networks. With continued exposure, children and parents may get more used to the changed health behaviours, and it may become easier for them to adopt them outside of the school setting. Spillover effects may be more likely for interventions that aim at changing the whole environment/system, like whole-school approaches, as compared to educational interventions, because whole-school approaches focus on making multiple changes (physical environment, school policy, community involvement, parental involvement etc.) to induce a ‘cultural shift’ among children, teachers, and parents. The transfer of behavioural changes is also influenced by environmental circumstances (e.g. beliefs, knowledge, habits in the household). “Provided that intervention fidelity doesn’t change, effects can become more favourable because children and parents get more used and acquainted with the behaviour change” “This particularly holds for young children that are exposed for a long time period”
3. *Personal factors*	*Context:* personal characteristics (sex, age, family characteristics, psychosocial health, and physical health) *Mechanisms:* decreased negative feelings with participating in sports, improved motoric abilities and fitness levels making exercising easier. *Outcome:* potential sustained relative effects Description: In addition to getting used to new lifestyle behaviours, the interventions might have an impact on other internal/psychosocial mechanisms (e.g. self-regulation, ‘not feeling comfortable’ with participating in sports). In addition, interventions might influence children’s motoric abilities, and fitness levels. These impacts can potentially contribute to some sustained effects on behaviour changes and BMI z-scores.

###### Constant exposure-effect scenario

Most experts indicated that the observed relative effects (after 2 years) would stay about the same when exposure is maintained during the primary school period (mentioned by N = 8 respondents).

###### Household multiplier scenario

Some experts anticipated that prolonged exposure (longer than the observed 2 years) during the primary school period and new learned behaviours might lead to behaviour changes within the household (mentioned by N = 3 respondents). This would probably lead to more favourable results as compared to the observed 2-year effects.

###### Personal factors

Last, experts indicated that in addition to the constant effect-exposure scenario, internal/psychosocial mechanisms (e.g. reducing feelings of ‘not feeling comfortable’ with participating in sports) and physical mechanisms (e.g. increase in muscle mass) could contribute to sustained effects (mentioned by N = 4 respondents).

##### 3.2.1.2 Phase 2: After the primary school period

###### Uncontrolled environment scenario

Nearly all respondents expected an effect decay after the primary school period (mentioned by N = 10 respondents). It was anticipated that the transition to secondary school would lead to a disruption of children’s pre-existing acquired health behaviours.

###### Household maintainer scenario

One expert indicated that the relative effects could be sustained via improved health behaviours in the household (Table 3).

**Table 3:** Expert scenarios regarding the unobserved effects after the primary school period.

Period: After the primary school period, 12 up to 20 years of age	
Uncertainty: Uncertainty on the (unobserved) relative effect after intervention exposure	
*See Appendix 6 for a selection of relevant literature.*	
Scenario	Description
1. Uncontrolled environment	*Context:* environment (behaviours and facilities in the household, schools, community, neighbourhood, influence of peers), personal characteristics (age, sex, future personal characteristics) *Mechanism:* influence of the environment (copying behaviours, facilitating/hindering behaviours through facilities), increased autonomy, return to previous experiences (behaviours) *Outcome:* decay of the relative effect, potential effects at older ages. Description: The effect maintenance after the primary school period is uncertain. After the primary school period, children will be exposed to an uncontrolled environment, which affects their lifestyle behaviours. Important factors in this environment such as behaviours of peers and parents (e.g. family routines, peer pressure), circumstances and physical characteristics at school (e.g. foods provided at school, proximity to supermarkets) and at home (e.g. availability of foods) may affect the effect maintenance. In this ‘obesogenic environment’, it is difficult to maintain behaviour changes. In secondary school, sport participation rates decline, and dietary behaviours become unhealthier. In addition, children become more autonomous, and peer influences of (non-exposed) peers will increase. All together, the relative intervention effects between the intervention and control group will probably decrease. The effects will decay within the first year of secondary school, and will then reach a plateau level. Potentially, children may adopt the behaviours changes again in young adulthood when starting a family life or career. At this time point, individual circumstances change which lead to deliberately considering health behaviours after a period of habit discontinuity. The evidence base on long-term effect maintenance is poor, and the extent of effect maintenance is hard to predict, because of the multifactorial aspects during the teenage and adolescence period. “The protected school environment is no longer present. A large proportion of the intervention effect will therefore decay.” “The differences in health behaviours between children from intervention groups and control groups will attenuate” “Children from intervention groups will adopt more unhealthy behaviours due to and group behaviour” “Continuation of lifestyle interventions at secondary schools is needed to generate long-term effects.”
2. Household maintainer	*Context:* environment (behaviours and facilities in the household, schools, community, neighbourhood, influence of peers, marketing), intervention exposure (components, intensity, duration) *Mechanisms:* sustained behaviour changes in the household *Outcome:* maintained or increased relative effects Description: Long-term exposure to health behaviours in the school setting can influence the household setting in which children grow up (see household multiplier scenario). When effects transfer to the household (see household multiplier scenario), it is also likely that they will be (somewhat) maintained. The effect transfer particularly pertains to continued interventions with a combination of nutrition and diet and extensive parental involvement, as transfer of dietary behaviours is most likely. The transfer of health behaviours to the household may be disproportionally distributed across family (socioeconomic) backgrounds with potentially disadvantaging low socioeconomic status groups. “According to my vision, the circumstances to which children are exposed during the primary school period will have impact on the household-setting”

The respondents highlighted that numerous characteristics in the environment (e.g. facilities in the neighbourhood, knowledge and beliefs, marketing, media, and policy factors) influence health behaviours and BMI. Most factors will affect children from both the control and intervention groups, and the relative effects will therefore not change greatly. Some experts indicated that it was too difficult for them to make quantitative estimations, due to the lack of specific expertise, and/or the multifactorial nature of the uncertainties (N = 4 experts, N = 2 interviews). Quantitative estimates for the scenarios ‘personal factors’ were not further quantitatively specified as they were considered too multifactorial by the respondents.

#### 3.2.2 Relative effects

Three different combinations of scenarios were mentioned.

A) **The constant exposure-effect scenario and the uncontrolled environment scenario** (mentioned by N = 8 respondents).
B) **The household multiplier scenario and the uncontrolled environment scenario** (mentioned by N = 2 respondents).
C) **The household multiplier scenario and the household maintainer scenario** (mentioned by N = 1 respondent).

**Fig. 3:**
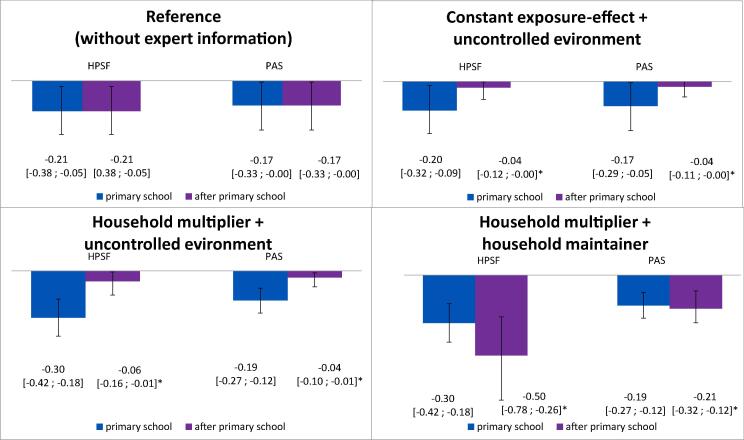
Title: Combination of uncertainty scenarios and corresponding BMI-effects [95% confidence interval]. Notes: HPSF = Healthy Primary School of the Future, PAS = Physical Activity School * statistically significant effect (p ≤ 0.05). The BMI-effects during the primary school period in the reference scenario are based on Bartelink et al. (2019) (Bartelink et al., 2019a).

The relative reductions in BMI during and after the primary school period are provided in Fig. 3.

## 4 Discussion

This study aimed to model plausible BMI trajectories after exposure to HPSF and PAS until early adulthood. Relying on the 2-year observed effects resulted in a relative effect of −0.21 (HPSF) and −0.17 kg/m^2^ (PAS). Experts indicated that intervention effects could be maintained during the primary school period (constant exposure-effect scenario) or become more favourable due to the adoption of behaviour changes in the household (household multiplier scenario). After the primary school period, effects could decay (uncontrolled environment scenario) or be maintained due to sustained behaviour changes in the household (household maintainer scenario). The smallest relative effects were found under a constant exposure-effect (primary school) and uncontrolled environment scenario (after primary school) (mean difference at 20 years of age: −0.04 kg/m^2^ [HPSF and PAS vs. control]. The highest relative effects corresponded to a household multiplier (primary school) and household maintainer scenario (after primary school) (mean difference at 20 years of age: −0.50 kg/m^2^ [HPSF vs. control], −0.21 kg/m^2^ [PAS vs. control]).

### 4.1 Study design

The quasi-experimental study design can be seen as a limitation, because the three groups (control schools, HPSF, and PAS) were not fully comparable due to the lack of randomization. At baseline, children at control schools had a lower SES-score and a higher BMI. Bartelink et al. (2019a) controlled for these differences when studying the 2-year BMI effects (Bartelink et al., 2019a). In the current study, we also controlled for SES and stratified the analysis by sex.

### 4.2 BMI trajectories from childhood to adulthood

In the current study, model parameters for the BMI trajectory of primary-school based children are used for extrapolating BMI trajectories during adolescence. Theoretically, this approach is suboptimal when one aims to accurately resemble the variation in BMI trajectories, because BMI growth and variability is different for adolescents as compared to primary school aged children. We observed that the fitted values of the models were not fully comparable to the results of the Fifth Dutch Growth Study, which is probably due to differences in the study population (children from the Parkstad region vs. general Dutch population) and secular trends (2015–2018 vs. 2009) (Schönbeck et al., 2011). Instead of the face validity criterion, databased criteria (maximum difference [%] between estimated and observed values) could be used to further improve the predictive ability of the BMI trajectory models for modelling BMI-values at older ages. We aimed to adequately capture the general BMI trajectory for children during main transitions in childhood. Lifecourse patterns in health behaviours and BMI are, however, complex and highly variable among individuals, which may not have been fully captured in the current study.

### 4.3 Expert judgement

At least six experts were invited for participation in accordance to recommendations by Cooke et al. (2006) (Cooke and Probst, 2006). The different backgrounds from health promotion experts, health economists, and epidemiologists led to the discovery of different scenarios. Not all participants felt confident with making quantitative estimations due to their unfamiliarity with the relative effects of primary school-based lifestyle interventions on BMI or due to the multifactorial nature of effect maintenance and secular trends. Using intermediate outcome measures, like the increase in minutes of moderate physical activity or calorie intake, instead of BMI, may potentially aid in making estimations on relative effects. Overall, we feel that integrating expert elicitation into the extrapolation of databased statistical models is a first step towards assessing the impact of effect maintenance on the intervention’s benefits in a more valid (expert informed estimates vs. uninformed scenario analyses) and informative manner.

### 4.4 Relative effectiveness of HPSF and PAS in childhood and adolescence

Regarding the primary school period, most experts anticipated constant relative effects. Bartelink et al. (2019) found that the BMI reductions in children at HPSF and PAS were smaller in the second year as compared to the first year of intervention, while the relative effects became larger due to the BMI growth at control schools. A decreased enthusiasm was mentioned as a potential explanation for the smaller intervention effects in the second year (Bartelink et al., 2019a). Analysis of the 4-year effects will be carried out to investigate whether the effects in the second year are sustained. Another anticipated scenario was the uptake of lifestyle changes in the household. The 2-year quantitative analysis did not show an overall effect for physical activity and dietary behaviour at home, but more favourable effects in physical activity at home were found for children with a higher SES background (Bartelink et al., 2019c). Further research could focus on the transfer of effects to the household and the impact on effect maintenance during and after the primary school period. Follow-up research in secondary schools is of utmost need to investigate the effect maintenance of primary school-based lifestyle interventions into adolescence and young adulthood.

### 4.5 Relative effectiveness of HPSF and PAS in adulthood

It may be difficult for readers to understand the impact and consequences of our findings as the uncertainty is only expressed in terms of the intermediate BMI outcome. In a modelling study, we will estimate the impact of the different scenarios on the long-term health and economic impacts of HPSF and PAS (e.g. healthcare cost savings, years lived with overweight). In order to provide more insight on the relevance of our findings, we converted the results on BMI into the effects on excess calorie intake, which is another intermediate outcome but may be easier to interpret. Wang et al. (Wang et al., 2012) and Plachta-Danielzik et al. (Plachta-Danielzik et al., 2008) calculated that children’s excess energy intake should be reduced by 41 kcal/day and 27–58 kcal/day in order to meet the goals for childhood obesity prevention in the US and Germany, respectively (Plachta-Danielzik et al., 2008, Wang et al., 2012). By applying the same mathematical model, we calculated that the relative effects for HPSF (0.21 kg/m^2^ in the reference scenario and 0.50 kg/m^2^ in the household multiplier and maintainer scenario) translated to a reduction of 17 and 40 kcal/day for an 8-year old boy, respectively.

## 5 Conclusions

The mixed-methods approach proved to be useful for unravelling uncertainty, for identifying evidence gaps, and for providing inputs for estimates on medium and long-term intervention benefits. The specification of uncertainty on intervention effect maintenance is a first step towards better informing adoption decisions on primary school-based lifestyle interventions. Future research should focus on long-term observations to reduce uncertainty.

## Declarations

### Ethics approval and consent to participate

The need for ethical approval has been waived by the Medical Ethics Committee Zuyderland in Heerlen (MEC 14-N-142). All participants were required to complete an informed consent form in accordance with the Declaration of Helsinki, signed by both parents/caregivers, and by the children in case they are 12 years or older. The study protocol has been registered in the database ClinicalTrials.gov (NCT02800616). The study was retrospectively registered in the ClinicalTrials.gov database on 14 June 2016 (NCT02800616).

### Consent for publication

Not applicable.

### Availability of data and materials

The data that support the findings of this study were collected as part of the ‘Healthy Primary School of the Future’ quasi-experimental study. Data collection will take place until 2019 to study the effects after 4 years of exposure. Data will become available following article publication on the 4-year effects and potential other comparative studies in the Netherlands.

### Funding

This study was funded by the Limburg provincial authorities, Project Number 200130003, by Friesland Campina, Project Number LLMV00, and by Maastricht University. None of the funding bodies had a role in the design of the study or the writing of this manuscript. Nor will the funding bodies have a role in the future data collection, analysis, interpretation of data, and the writing of publications.

## Authors contributions

MO, MAJ, and SJ designed the study. MO and SJ performed the statistical analyses. MO conducted the interviews and MO and DDBG were responsible for processing and interpreting the results of the interviews. MO wrote the manuscript with input from all authors (MO, SJ, DDBG, AVG, HB, OCPVS, and MAJ). All authors read and approved the final manuscript.

## Declaration of Competing Interest

The authors declare that they have no known competing financial interests or personal relationships that could have appeared to influence the work reported in this paper.
