# Supplementary material for: BMI trajectories after primary school-based lifestyle intervention: Unravelling an uncertain future. A mixed methods study
Source: Prev Med Rep. 2021 Jan 7;21:101314. doi: 10.1016/j.pmedr.2021.101314 (PMC7841358; doi:10.1016/j.pmedr.2021.101314)
Supplement: Supplementary data 1 [file mmc1.docx]

Appendix A. **Flowchart**

**Figure A1. Flowchart of children participating in the measurements [15].**

| **Baseline**  Total participating children: n=1403 (60.3% of all children) | | |
| --- | --- | --- |
| **Selection for the current study***  Participating children in classes 1-7: n=1255 | | |
| **HPSF (n=361) 2 schools** | **PAS (n=408) 2 schools** | **Control (n=486) 4 schools** |
|  |  |  |
| **One year follow up (Year 1)**  Total participating children: n=1489 (60.7% of all children) | | |
| **Selection for the current study**  Participating children in classes 2-8: n=1455  Newly included (n=264); Drop-out** (n=64) | | |
| **HPSF (n=469)**  New included: n=132  Drop-out: n=24 | **PAS (n=428)**  New included: n=33  Drop-out: n=13 | **Control (n=558)**  New included: n=99  Drop-out: n=27 |
|  |  |  |
| **Two year follow up (Year 2)**  Total participating children: n=1470 (61.7% of all children) | | |
| **Selection for the current study**  Participating children in classes 3-8: n=1323  Newly included (n=158); Drop-out*** (n=290) | | |
| **HPSF (n=432)**  New included: n=44  Drop-out: n=81 | **PAS (n=376)**  New included: n=38  Drop-out: n=90 | **Control (n=515)**  New included: n=76  Drop-out: n=119 |
|  |  |  |
| **Total participating children in study period: n=1974** | | |
| **Selection for the current study** Enrolled at school at baseline: n=1676 **** | | |
| **HPSF (n=537)** | **PAS (n=478)** | **Control (n=661)** |
| **Selection for the mixed model analyses: n=1647**  Complete data on covariates & BMI data on at least one time point: n=1647 | | |
| **HPSF (n=525)** | **PAS (n=473)** | **Control (n=649)** |

**Notes:** HPSF = Healthy Primary School of the Future, PAS = Physical Activity School, Year 1= after 12 months; Year 2 = after 24 months.

* All children from study year one to eight (age 4 to 12) enrolled at the eight participating schools were eligible to participate in the study, which is internationally comparable to two years of Kindergarten and six grades.
** Reasons drop out Year 1: switched to other included school (n=2), other reasons e.g. moved out or actively stopped participation (n=62).

*** Reasons drop out Year 2: finished school (n=228), switched to other included school (n=17), other reasons e.g. moved out or actively stopped participation (n=45).

**** Children who joined the study at year1 one or year2 were included when they were already enrolled in the participating school at baseline.

Table A1. Characteristics of the original study sample (N=1676) at baseline (T0) [13].

|  |  | **Total (N=1676)** | |  | **HPSF (N=537)** | **PAS (N=478)** | **Control (N=661)** |
| --- | --- | --- | --- | --- | --- | --- | --- |
|  |  | ***N*** | ***Missing (%)^π^*** | ***% / Mean (±SD)*** | ***% / Mean (±SD)*** | ***% / Mean (±SD)*** | ***% / Mean (±SD)*** |
| Sex (% boys) |  | 1676 | 0 (0%) | 47.4% | 47.7% | 47.3% | 47.2% |
| Age (years) |  | 1676 | 0 (0%) | 7·5 (±2·16) | 7·6 (±2·16) | 7·4 (±2·22) | 7·6 (±2·13) |
| Ethnicity (% Western)^¥^ | | 1016 | 660 (39.4%) | 94.1% | 93.0% | 96.0% | 93.4% |
| SES (%)^Ф^ | *Lowest tertile* | 1673 | 3 (0.2%) | 31.3% | 31.8% | 15.9% | 42.1% |
|  | *Middle tertile* | ·· | | 34.5% | 29.2% | 43.4% | 32.4% |
|  | *Highest tertile* | ·· | | 34.2% | 39.1% | 40.8% | 25.5% |
| BMI z-score ^§^ |  | 1109 | 567 (33.8%) | 0·135 (±1·02) | 0·051 (±1·01) | 0·092 (±0·95) | 0·232(±1·07) |
| BMI | Total | 1109 | 567 (33.8%) | 17·04 (±2·55) | 16·80 (±2·39) | 16·85 (±2·31) | 17.37 (±2.81) |
|  | Age 5-7 | ·· | | 16.15 (±1.78) | 16·23 (±1·85) | 16·15 (±1·58) | 16·10 (±1·90) |
|  | Age 8-10 | ·· | | 17.41 (±2.67) | 16·84 (±2·32) | 17·02 (±2·26) | 18·08 (±3·00) |
|  | Age 11-13 | ·· | | 18.28 (±3.10) | 18·32 (±3·27) | 18·27 (±3·20) | 18·27 (±2·92) |
| Overweight & obesity ^*§^ | Total | 1109 | 567 (33.8%) | 19.9% | 16.5% | 17.9% | 24.1% |
| Overweight ^*^ |  | ·· | | 15.9% | 13.1% | 16.2% | 17.7% |
| Obesity ^*^ |  | ·· | | 4.0% | 3.4% | 1.7% | 6.4% |

**Notes:** BMI = body mass index, HPSF = Healthy Primary School of the Future, PAS = Physical Activity School, SD = standard deviation, SES = socioeconomic status.
^¥^ Information on children’s ethnicity was collected from annual parental questionnaires. Ethnicity, being native background, Western background or a Non-Western background was based on the country of birth of both parents. Ethnicity was subsequently divided into Western (including native background) and non-Western background [15].
^Ф^ Socioeconomic status (SES) was based on information from a parent-reported questionnaire, and calculated as the mean of standard scores on maternal education, paternal education, household income (adjusted for household size), and neighbourhood SES score (latter derived from the Netherlands Institute for Social Research). ^§^ Pearson chi-square and ANOVA tests indicated statistically significant differences between the groups (BMI-z: p=0.034, weight status: p=0.006), see Bartelink et al. (2019) [15].
^*^ International Obesity Task Force (IOTF) cut-off values for childhood overweight and obesity.
^π^ Missing data at baseline were due to later participation in the study, incomplete information on ethnicity and SES, which were obtained from a parental questionnaire, no measurement of height/weight in the first grade.

Appendix B. **Elicitation protocol**

The expert elicitation followed the seven steps as described by Knol et al. (2010), and reporting was in accordance to the COREQ guidelines (reporting for qualitative studies).^1,2^

Characterization of uncertainties and typology of uncertainties
Empirical studies that evaluate childhood lifestyle interventions generally have a short duration of 1 to 4 years, and do not go beyond childhood. Population health models, are available for estimating the long-term health and cost impacts of lifestyle interventions as they link risk factors to diseases, morbidity, mortality, and costs, but generally start from adulthood onwards. To estimate the long-term health and cost impacts of childhood lifestyle interventions, the gap between childhood (observation range of empirical studies) and adulthood (captured in population health models) has to be filled. Filling this gap comes with uncertainty. The aim of the expert elicitation is to gather informed opinions of experts on the unobserved effects, effect maintenance, and the associated uncertainty.
We distinguish two uncertainties that are associated with plugging the evidence gap. The first type of uncertainty pertains to the primary school period (4-12 years of age corresponding to the Dutch setting). Children are still exposed to the intervention, but the effects are partially or no longer observed within the empirical study. The second type refers to the uncertainty on the relative effect after the primary school period when exposure to the intervention has ended.

Scope/format of elicitation

The elicitation will be performed by means of a semi-structured interview, performed face-to-face or by telephone (MO), and will follow a pre-developed interview guide. It is aimed to elicit experts’ views on the future trends in effect maintenance and the underlying mechanisms, as well as eliciting quantitative estimates on the future relative effects and the corresponding uncertainty (indicated by a mean estimate and an uncertainty distribution). The interviewer (MSc, female) is trained as a health scientist with expertise in health technology assessment. Expert’s views will be elicited in individual interviews where possible, to obtain individual views on the uncertainties, as recommended by Knol et al. (2010).^1^ The team will review whether additional group elicitation may be useful for sharing knowledge and for better appreciation of different disciplinary viewpoints.

1. Selection of experts
   In accordance to the recommendations of Cooke et al. (2006) we will invite at least 6 experts.^3^ It is aimed to have an information-rich and diverse panel. Therefore, we strive to get a mixed panel of experts (health promotion specialists, epidemiologists, cost-effectiveness researchers, dieticians/paediatricians) from different universities/institutions. Experts will be selected based on purposive sampling (N=11). A relationship with some of the experts was established prior to study commencement (N=4). A total of six experts are familiar with the Healthy Primary School of the Future initiative. Data saturation will be determined by the point at which no new unique themes are introduced (inductive thematic saturation).

Design of elicitation protocol
We will ask experts about their views on the presence of the two uncertainties (qualitatively), and subsequently ask to define a plausible parameter space to reflect the amount of uncertainty. As background information for the quantitative part, we will show the 2-year relative effects of HPSF and PAS vs. control schools. Participants will be asked to specify the unobserved relative effects during and after the primary school period. Graphical displays from the interactive MATCH Uncertainty Elicitation Tool will be used in which experts can specify the uncertainty around the anticipated mean relative effect. Participants are asked to specify the plausible parameter space based on the Roulette method (SHELF elicitation template), in order to retrieve the prior mean and variances for the model parameters. We will make use of the Roulette method, because this method is experienced as most intuitive by participants.^4^ Participants are asked to provide motivation for their answers in order to reduce biases and increase the interpretation of results and potential outliers. No seed variables were included in the interview protocol (actual values are known to analysts but unknown to experts) to minimize the burden and time investments for participants ( a minimum of 8 to 10 variables is required to use the seed variables for the analysis).^1^ The MATCH Elicitation software (MATCH tool) will be used for recording of quantitative answers, and detailed notes are taken for the qualitative answers during and after the interview. The expert elicitation protocol will be piloted among two experts.

Preparation of the elicitation session

Experts will receive an email with background information and the overall study objective, and are asked to participate in the study. During the interview (of about 45 minutes), the experts will receive additional information on the study aim, background of the researcher (PHD candidate researching cost-effectiveness on the Healthy Primary School of the Future initiative), the interventions, and the elicitation procedure.

**Background information on the problem**School-based lifestyle interventions have the potential to generate outcomes within and beyond childhood, due to the likely persistence of health behaviours and excess weight over time, and because excess weight is associated with considerable morbidity, mortality, and cost impacts from young adulthood onwards. Ignoring these long-term outcomes most likely underestimates the value of school-based lifestyle interventions. In order to employ obesity models for estimating the long-term cost-effectiveness of school-based lifestyle interventions, it is key to plug the evidence gap between the time horizon of empirical studies and young adulthood. Estimating outcomes for the unobserved period comes, however, with uncertainty.

**Goal**The aim of this study is to model the BMI trajectories for children (4-12 years of age) who participated in the ‘Healthy Primary School of the Future’ project up to young adulthood (20 years of age). BMI trajectories are modelled ‘the Healthy Primary School of the Future’ (HPSF) and the ‘Physical Activity School’ (PAS), and for a control group without any intervention. We aim to obtain information on the extent of the uncertainty associated with plugging this evidence gap. We will therefore ask you some questions on the long-term unobserved effects and the uncertainty around these values.

**Uncertainties**

Most of the empirical studies on childhood lifestyle interventions include a school cohort, with children from varying ages (e.g. children in the age range 4-12 years), which are followed over the time horizon of the empirical study with an average duration of 1-4 years. Given the limited time horizon of most empirical studies, the weight trajectories of individual children are usually partially observed during the primary school period. We distinguish two uncertainties that are associated with plugging the evidence gap between. The first type of uncertainty pertains to the primary school period (4-12 years of age corresponding to the Dutch setting). Children are still exposed to the intervention, but the effects are partially or no longer observed within the empirical study. The second type refers to the uncertainty on the relative effect after the primary school period when children when exposure to the intervention has ended (effect maintenance).

**Elicitation procedure**

In an expert interview, the templates of the Sheffield Elicitation Framework (SHELF) and the web-based MATCH Uncertainty Elicitation Tool will be used for presenting questions and to guide the elicitation of uncertainty information.^4^ The interview will motivate the respondents to explain and provide rationale for their answers. Participants will be asked how difficult it was for them to answer the questions, and which background/expertise they found useful for answering the questions.

We will ask you to provide your view on the uncertainties that I just explained. I provided background information on the problem and the aim of this interview. I will provide you with some information on childhood BMI development and provide relevant data. You are also invited to add relevant literature. I will then ask you to make some estimates. We will use this to explore the different views on the uncertainty of extrapolating childhood weight trajectories. I will ask you to define a range of plausible values, and which values you consider more or less plausible. We will ask you to provide your personal view. We will interview multiple experts to obtain multiple views on this topic.

We will show you a grid comprising a number of columns and a range of possible values of X. These columns are called bins. We will ask you to specify the bin boundaries, which indicate the lowest and the highest plausible values of X. You will get a number of counters; each of them represents an amount of probability. You will receive 10 counters, so each of them represents a probability of 0.1 (10%). You are asked to place the counters in the bins on your grid, to specify your beliefs about X. Please check if you have used all your counters.

**Questionnaire**1A. Are the observed relative effects of HPSF and PAS after 2 years, representative for the entire primary school period if children are exposed to the interventions during the entire primary school period? Why?

1B. Realistically, what is the lowest plausible value for the relative effect of HPSF versus control schools at age 12 (end of primary school period)?
Realistically, what is the highest plausible value for the relative effect of HPSF versus control schools at age 12 (end of primary school period)?
Please place the counters on the presented grid to represent your beliefs regarding the uncertainty on the relative effect. *Idem for PAS.*

2A. Are the observed relative effects representative for the period after the primary school period (corresponding to age 13 up to 20 years of age) when children are no longer exposed to the primary school interventions? Why?

2B. Effect maintenance or decay is expressed in percentages: 0% (no sustained effects) – 100% (all effects maintained).
Realistically, what is the lowest plausible value for the effect maintenance of HPSF versus control schools at age 20 (end of primary school period)?
Realistically, what is the highest plausible value for the effect maintenance of HPSF versus control schools at age 20 (end of primary school period)?
Please place the counters on the presented grid to represent your beliefs regarding the uncertainty on the effect maintenance.
*Idem for PAS.*

Elicitation of expert judgments

To capture the variation in the experts’ set of assumptions, qualitative answers will be analysed after the interviews with thematic content analysis (MO and DDBG). Mechanisms of effect maintenance will be specified using the context-mechanism-outcome configuration of the realist evaluation method.^5^ Scenarios are drafted for the effect maintenance during the primary school period (1), and after the primary school period (2) (MO and DDBG).
A member check questionnaire (written) will be used to verify and validate the interpretation of experts’ answers. Based on a summary of the participant’s answers (drafted by MO), participants are asked whether they recognize their answers, and whether something is missing or unclear. In addition, critical peer review between two authors (MO and DDBG) will be performed to check the interpretation and aggregation of outcomes in scenarios. The synthesized information will also be part of the member check (synthesized member check).^6^  Participants are asked about whether they recognize their views in one of the scenarios, if something is missing, and are asked to indicate the likelihood of each of the scenarios. This information will be used to update the synthesized results.

Possible aggregation and reporting
Differences in judgements between experts might stem from different background information on which the experts make their judgement or from different schools of thought.^7^ Diversity of expert views itself carries valuable information and should be part of the open reporting of the study results. We will examine whether disparate views are present. We will do this via first synthesizing the views on the uncertainties that emerge from the qualitative questions. Scenarios will be described, and participant quotations will be presented. Due to limitations in time, and to keep the interview concise, we will not include seed variables for determining expert weights. Instead, we will use linear pooling, in accordance to others.^7,8^

Appendix C**. Model details primary school period**

Table C1. Parameter estimates for children at control schools with an average SES, with model 1 and model 2 (outcome = BMI).

|  | **Boys** | | | **Girls** | | |
| --- | --- | --- | --- | --- | --- | --- |
| **Parameter** | **Estimate ^1^** | **LL** | **UL** | **Estimate** | **LL** | **UL** |
| **Model 1** |  |  |  |  |  |  |
| *Fixed effects* |  |  |  |  |  |  |
| Intercept | 19·133 | 16·221 | 22·552 | 16·068 | 13·414 | 19·240 |
| Linear slope for age | -1·922 | -2·373 | -1·169 | -0·502 | -1·134 | 0·485 |
| Quadratic slope for age (age ^2) | 0·299 | 0·153 | 0·493 | 0·110 | 0·004 | 0·259 |
| Cubic slope for age (age ^3) | -0·098 | -0·012 | -0·006 | -0·003 | -0·006 | 0·001 |
| HPSF | 1·031 | 0·179 | 2·222 | 0·462 | -0·190 | 1·419 |
| PAS | 0·551 | -0·190 | 1·603 | 0·514 | -0·154 | 1·488 |
| Linear slope for age *HPSF | -0·156 | -0·220 | -0·062 | -0·105 | -0·159 | -0·023 |
| Linear slope for age *PAS | -0·105 | -0·176 | -0·002 | -0·075 | -0·135 | 0·015 |
| Low SES | 0·386 | -0·003 | 0·923 | 0·173 | -0·146 | 0·632 |
| High SES | -0·117 | -0·404 | 0·297 | -0·408 | -0·598 | -0·112 |
| **Model 2** |  |  |  |  |  |  |
| *Fixed effects* |  |  |  |  |  |  |
| Intercept | 13·646 | 12·815 | 14·530 | 13·844 | 12·914 | 14·841 |
| Linear slope for age | 0·345 | 0·286 | 0·410 | 0·356 | 0·295 | 0·425 |
| Post-change linear slope for age ≥ 6 years | 0·083 | -0·539 | 0·829 | 0·201 | -0·497 | 1·050 |
| Post-change linear slope for age ≥ 9 years | 0·459 | -0·045 | 1·050 | 0·501 | 0·029 | 1·058 |
| HPSF | 0·124 | -0·224 | 0·529 | -0·095 | -0·449 | 0·325 |
| PAS | 0·074 | -0·264 | 0·466 | 0·189 | -0·197 | 0·644 |
| Post-change slope ≥ 6 years *HPSF | -0·167 | -0·435 | 0·141 | -0·150 | -0·429 | 0·175 |
| Post-change slope ≥ 6 years *PAS | -0·185 | -0·437 | 0·109 | -0·171 | -0·449 | 0·155 |
| Post-change slope ≥ 9 years *HPSF | -0·137 | -0·323 | 0·079 | -0·071 | -0·252 | 0·140 |
| Post-change slope ≥ 9 years *PAS | -0·181 | -0·368 | 0·079 | -0·247 | -0·431 | -0·031 |
| Low SES | 0·265 | -0·014 | 0·585 | 0·132 | -0·157 | 0·468 |
| High SES | -0·076 | -0·314 | 0·202 | -0·339 | -0·564 | -0·072 |

**Notes:** HPSF = Healthy Primary School of the Future, PAS = Physical Activity School, Post change linear slope for age ≥ 6 years = dummy variable for age of 6 or older (1=≥6 years, 0=<6 years), Post change linear slope for age ≥ 9 years = dummy variable for age of 9 or older (1=≥9 years, 0=<9 years), low SES = low socioeconomic background, high SES = high socioeconomic background.
^1^ All parameter estimates were back transformed from the lnBMI scale to the original scale (BMI).

**Table C2. Observed and predicted BMI values.**

|  | **Predicted values Model 1 control schools, average SES (N=214)** | | **Predicted values Model 2 control schools, average SES (N=214)** | | **Observed values at T0, T1, or T2 control schools, average SES (N=208)** | |
| --- | --- | --- | --- | --- | --- | --- |
|  | ***Mean [95% CI]*** | ***Median [IQR]*** | ***Mean [95% CI]*** | ***Median [IQR]*** | ***Mean [95% CI]*** | ***Median [IQR]*** |
| **Control school** |  |  |  |  |  |  |
| **Boys** |  |  |  |  |  |  |
| Age 5 | 15·32 [14·21 ; 15·99] | 15·27 [1·78] | 15·29 [14·19 ; 15·94] | 15·24 [1·75] | 15·26 [14·19 ; 15·82] | 15·30 [1·63] |
| Age 6 | 15·70 [14·32 ; 16·28] | 15·49 [1·96] | 15·66 [14·27 ; 16·22] | 15·45 [1·95] | 15·67 [14·26 ; 16·54] | 15·50 [2·28] |
| Age 7 | 15·75 [14·31 ; 16·43] | 15·27 [2·12] | 15·80 [14·36 ; 16·44] | 15·30 [2·07] | 15·82 [14·47 ; 16·34] | 15·17 [1·87] |
| Age 8 | 16·78 [15·31 ; 17·71] | 16·90 [2·40] | 16·79 [15·34 ; 17·72] | 16·89 [2·37] | 16·84 [15·56 ; 18·16] | 16·81 [2·60] |
| Age 9 | 17·02 [15·20 ; 18·24] | 17·14 [3·05] | 17·10 [15·31 ; 18·33] | 17·14 [3·02] | 16·95 [15·09 ; 18·38] | 16·93 [3·29] |
| Age 10 | 17·95 [15·72 ; 19·74] | 17·59 [4·01] | 17·97 [15·72 ; 19·65] | 17·61 [3·93] | 18·00 [15·71 ; 19·91] | 17·45 [4·20] |
| Age 11 | 18·79 [16·08 ; 21·31] | 17·93 [5·23] | 18·75 [16·03 ; 21·23] | 17·94 [5·21] | 18·74 [16·26 ; 20·60] | 17·70 [4·33] |
|  |  |  |  |  |  |  |
| **Girls** |  |  |  |  |  |  |
| Age 5 | 15·19 [14·36 ; 15·94] | 14·87 [1·58] | 15·16 [14·34 ; 15·91] | 14·83 [1·57] | 15·27 [14·42 ; 16·01] | 15·03 [1·59] |
| Age 6 | 16·27 [14·86 ; 16·62] | 15·71 [1·76] | 16·24 [14·84 ; 16·62] | 15·68 [1·78] | 16·19 [14·86 ; 16·69] | 15·42 [1·83] |
| Age 7 | 16·81 [14·97 ; 17·98] | 16·01 [3·02] | 16·83 [14·98 ; 17·98] | 16·04 [2·99] | 16·82 [14·91 ; 17·72] | 15·94 [2·81] |
| Age 8 | 17·30 [15·35 ; 18·04] | 16·50 [2·69] | 17·27 [15·31 ; 18·04] | 16·49 [2·72] | 17·34 [15·19 ; 18·22] | 16·56 [3·03] |
| Age 9 | 17·44 [15·88 ; 18·04] | 16·87 [2·15] | 17·52 [15·91 ; 18·17] | 17·00 [2·26] | 17·48 [15·83 ; 18·11] | 16·69 [2·28] |
| Age 10 | 17·75 [16·13 ; 18·56] | 17·42 [2·43] | 17·77 [16·14 ; 18·56] | 17·47 [2·41] | 17·70 [16·01 ; 18·48] | 17·23 [2·48] |
| Age 11 | 18·29 [16·52 ; 19·39] | 18·05 [2·87] | 18·24 [16·52 ; 19·37] | 18·05 [2·86] | 18·27 [16·56 ; 19·53] | 17·99 [2·96] |
|  |  |  |  |  |  |  |

**Notes:** BMI = body mass index, CI = confidence interval, IQR = interquartile range, SES = socioeconomic status.

**Figure C1. Histogram of observed (column 1), fitted values with model 1 (column 2), and fitted values with model 2 (column 3) for boys at control schools with an average SES.**


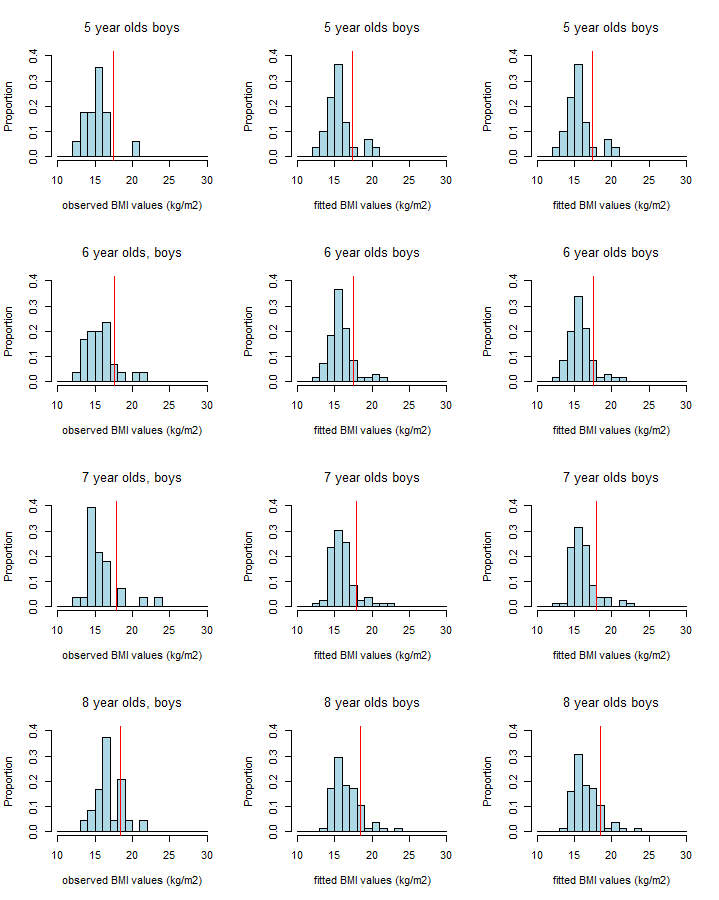


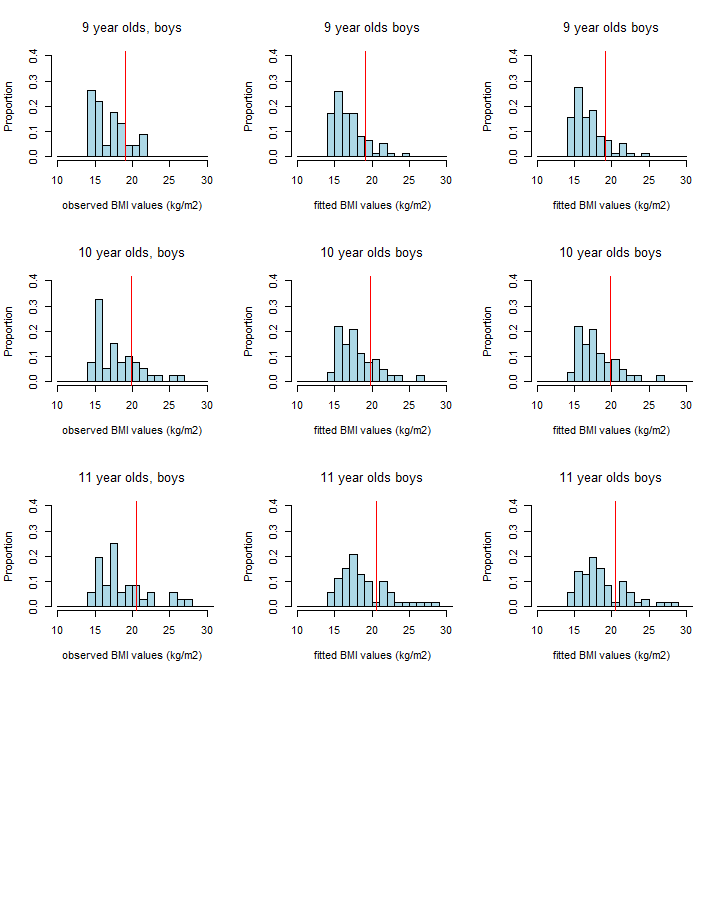


**Notes:** IOTF = International Obesity Task Force
red vertical line = IOTF cut-off points for childhood overweight and obesity (age- and sex-specific).^9^

**Figure C2. Histogram of observed (column 1), fitted values with model 1 (column 2), and fitted values with model 2 (column 3) for girls at control schools with an average SES.**


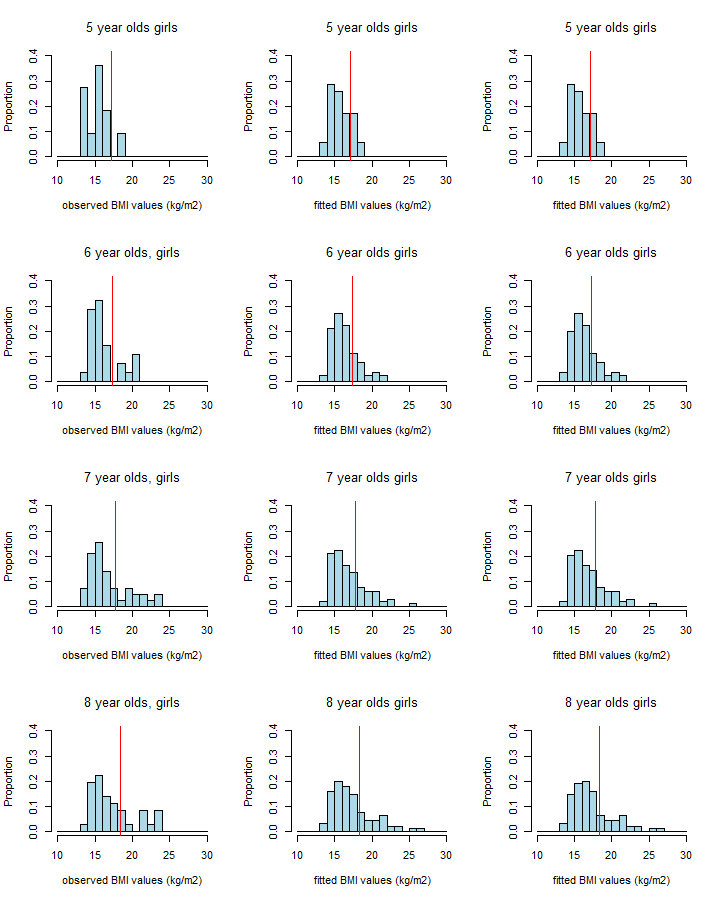


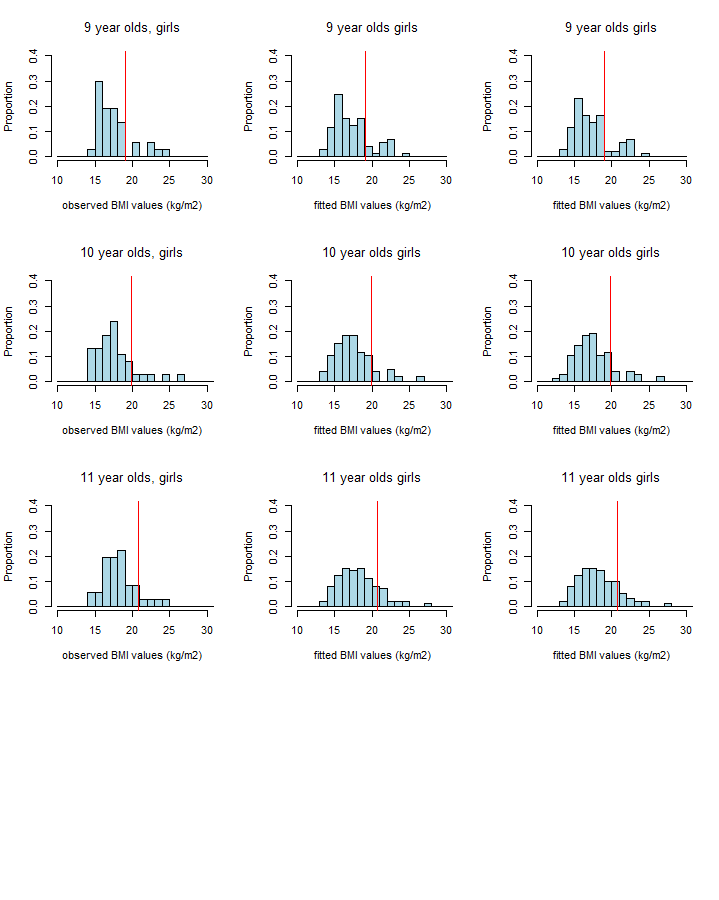


**Notes:** BMI = body mass index, IOTF = International Obesity Task Force
red vertical line = IOTF cut-off points for childhood overweight and obesity (age- and sex-specific).^9^

|  | **Predicted values Model 1 control schools, average SES** | | | **Predicted values Model 2 control schools, average SES** | | | **FDGS** |
| --- | --- | --- | --- | --- | --- | --- | --- |
|  | ***Mean [95% CI]*** | ***Median [IQR]*** | | ***Mean [95% CI]*** | ***Median [IQR]*** | | ***Median*** |
| **Boys** |  |  | |  |  | |  |
| Age 12 | 19·27 [16·57 ; 20·35] | 18·74 [3·78] | | 18·87 [16·19 ; 20·15] | 18·31 [3·96] | | 17·75 |
| Age 13 | 19·88 [16·81 ; 21·05] | 19·29 [4·24] | | 19·43 [16·41 ; 20·80] | 18·77 [4·39] | | 18·31 |
| Age 14 | 20·31 [16·94 ; 21·64] | 19·57 [4·70] | | 20·01 [16·61 ; 21·64] | 19·25 [5·03] | | 18·94 |
| Age 15 | 20·47 [16·79 ; 21·98] | 19·74 [5·19] | | 20·59 [16·89 ; 22·39] | 19·72 [5·50] | | 19·59 |
| Age 16 | 20·40 [16·49 ; 22·07] | 19·58 [5·58] | | 21·24 [17·10 ; 23·28] | 20·22 [6·18] | | 20·21 |
| Age 17 | 19·88 [15·85 ; 21·68] | 19·03 [5·83] | | 21·86 [17·35 ; 24·07] | 20·71 [6·73] | | 20·78 |
| Age 18 | 18·89 [14·84 ; 20·79] | 17·88 [5·96] | | 22·53 [17·56 ; 24·94] | 21·25 [7·38] | | 21·26 |
| Age 19 | 17·71 [13·70 ; 19·56] | 16·68 [5·86] | | 23·26 [17·85 ; 25·56] | 21·81 [7·72] | | 21·68 |
| Age 19 * | NA | NA | | 22·95 [17·98 ; 25·36] | 21·67 [7·38] | | 21·68 |
| Age 20 | 16·00 [12·12 ; 17·65] | 15·03 [5·53] | | 24·01 [17·99 ; 26·67] | 22·34 [8·67] | | 22·07 |
| Age 20 * | NA | NA | | 23·34 [18·37 ; 25·75] | 22·06 [7·38] | | 22·07 |
|  |  |  |  |  |  |  |  |
| **Girls** |  |  | |  |  | |  |
| Age 12 | 19·46 [16·86 ; 20·63] | 18·77 [3·77] | | 18·98 [16·53 ; 20·16] | 18·25 [3·64] | | 18·21 |
| Age 13 | 20·20 [17·26 ; 21·43] | 19·38 [4·17] | | 19·50 [16·77 ; 20·94] | 18·71 [4·17] | | 18·83 |
| Age 14 | 20·92 [17·66 ; 22·29] | 20·04 [4·63] | | 20·06 [17·07 ; 21·54] | 19·08 [4·47] | | 19·47 |
| Age 15 | 21·60 [17·98; 23·11] | 20·56 [5·14] | | 20·61 [17·31 ; 22·28] | 19·54 [4·97] | | 20·06 |
| Age 16 | 22·28 [18·29 ; 23·87] | 21·08 [5·57] | | 21·21 [17·57 ; 23·01] | 19·98 [5·44] | | 20·58 |
| Age 17 | 22·82 [18·53 ; 24·48] | 21·48 [5·95] | | 21·81 [17·86 ; 23·81] | 20·36 [5·94] | | 21·01 |
| Age 18 | 23·22 [18·58 ; 25·09] | 21·69 [6·51] | | 22·45 [18·20 ; 24·65] | 20·98 [6·45] | | 21·36 |
| Age 19 | 23·43 [18·56 ; 25·31] | 21·88 [6·75] | | 23·08 [18·37 ; 25·19] | 21·35 [6·82] | | 21·63 |
| Age 19 * | 23·49 [18·85 ; 25·36] | 21·96 [6·51] | | NA | NA | | 21·63 |
| Age 20 | 23·74 [18·57 ; 25·82] | 21·99 [7·25] | | 23·77 [18·76 ; 25·99] | 21·92 [7·24] | | 21·85 |
| Age 20 * | 23·71 [19·07 ; 25·58] | 22·18 [6·51] | | NA | NA | | 21·85 |
|  |  |  | |  |  | |  |

Appendix D**. Model details post-primary school period

Table D1. Observed and predicted BMI values.**

**Notes:** BMI = body mass index, CI = confidence interval, FDGS = Fifth Dutch Growth Study, IQR = interquartile range, NA = not applicable, SES = socioeconomic status.

* Calibration based on the trend in the Fifth Dutch Growth Study (see main text).

**
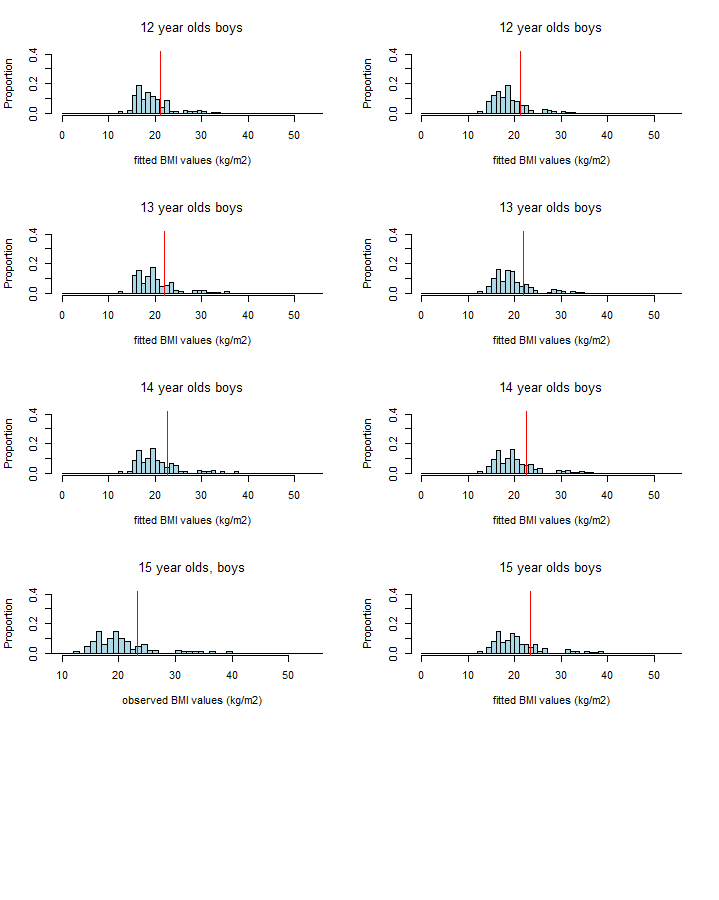
Figure D1. Histogram of fitted values with model 1 (column 1), and fitted values with model 2 (column 2) for boys at control schools with an average SES.**


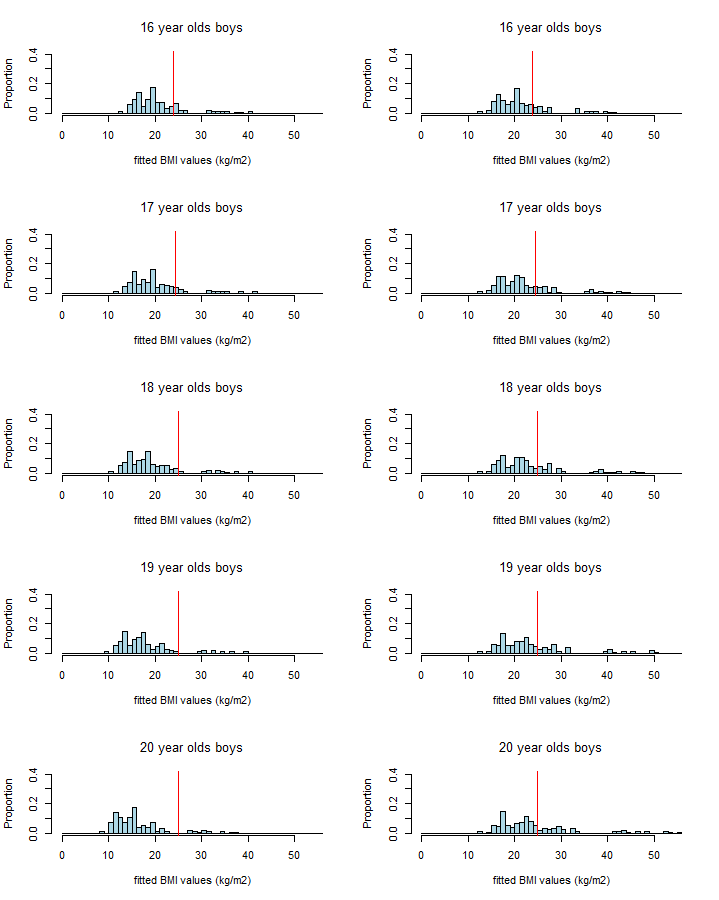


**Notes:** BMI = body mass index, IOTF = International Obesity Task Force
red vertical line = IOTF cut-off points for childhood overweight and obesity (age- and sex-specific).^9^

**
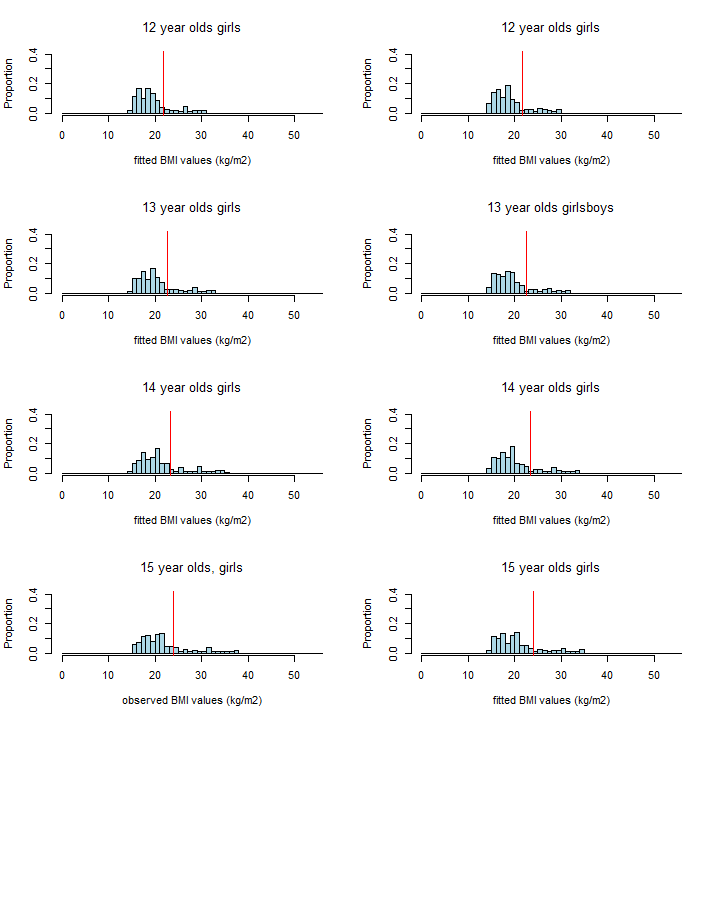
Figure D2. Histogram of fitted values with model 1 (column 1), and fitted values with model 2 (column 2) for girls at control schools with an average SES.**


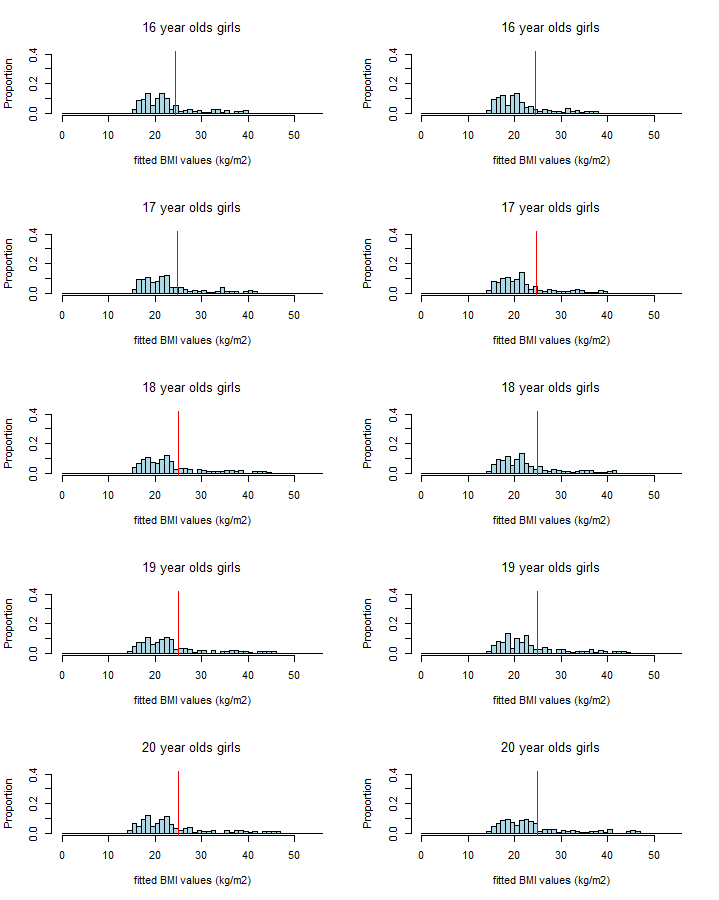


**Notes:** BMI = body mass index, IOTF = International Obesity Task Force
red vertical line = IOTF cut-off points for childhood overweight and obesity (age- and sex-specific).^9^

Appendix E**. BMI trajectories under different effect maintenance scenarios**

| **HPSF** | ***Control group*** | **Without expert information** | | **With expert information 1** Constant exposure-effect & uncontrolled environment | | **With expert information 2**  Household multiplier & uncontrolled environment ^π^ | | **With expert information 2**  Household multiplier & household maintainer  ^π^ | |
| --- | --- | --- | --- | --- | --- | --- | --- | --- | --- |
|  | ***Median*** | ***Median*** | ***[LL ; UL] ^¥^*** | ***Median*** | ***[LL ; UL]^¥^*** | ***Median*** | ***[LL ; UL]^¥^*** | ***Median*** | ***[LL ; UL]^¥^*** |
| **Boys** ^ǂ^ |  |  |  |  |  |  |  |  |  |
| Age 6 | 15·45 | 15·24 | [15·06 ; 15·40] | 15·25 | [15·13 ; 15·58] | 15·24 | [15·06 ; 15·40] | 15·24 | [15·06 ; 15·40] |
| Age 7 | 15·30 | 15·09 | [14·92 ; 15·25] | 15·10 | [14·99 ; 15·71] | 15·00 | [14·88 ; 15·13] | 15·00 | [14·88 ;15·13] |
| Age 8 | 16·89 | 16·68 | [16·51 ; 16·84] | 16·69 | [16·58 ; 16·81] | 16·59 | [16·47 ; 16·72] | 16·59 | [16·47 ; 16·72] |
| Age 9 | 17·14 | 16·93 | [16·76 ; 17·09] | 16·94 | [16·82 ; 17·05] | 16·84 | [16·72 ; 16·96] | 16·84 | [16·72 ; 16·96] |
| Age 10 | 17·61 | 17·40 | [17·22 ; 17·56] | 17·41 | [17·29 ; 17·52] | 17·31 | [17·19 ; 17·43] | 17·31 | [17·19 ; 17·43] |
| Age 11 | 17·94 | 17·73 | [17·56 ; 17·89] | 17·74 | [17·63 ; 17·86] | 17·65 | [17·52 ; 17·77] | 17·65 | [17·52 ; 17·77] |
| Age 12 |  | 18·10 | [17·92 ; 18·26] | 18·26 | [18·19 ; 18·30] | 18·24 | [18·14 ; 18·30] | 17·81 | [17·53 ; 18·05] |
| Age 13 |  | 18·56 | [18·39 ; 18·72] | 18·73 | [18·65 ; 18·77] | 18·71 | [18·61 ; 18·76] | 18·27 | [17·99 ; 18·51] |
| Age 14 |  | 19·04 | [18·86 ; 19·20] | 19·20 | [19·13 ; 19·24] | 19·18 | [19·08 ; 19·24] | 18·75 | [18·47 ; 18·99] |
| Age 15 |  | 19·51 | [19·34 ; 19·67] | 19·67 | [19·60 ; 19·71] | 19·65 | [19·55 ; 19·71] | 19·22 | [18·94 ; 19·46] |
| Age 16 |  | 20·01 | [19·84 ; 20·17] | 20·18 | [20·10 ; 20·22] | 20·16 | [20·06 ; 20·21] | 19·72 | [19·44 ; 19·96] |
| Age 17 |  | 20·50 | [20·32 ; 20·66] | 20·66 | [20·58 ; 20·70] | 20·64 | [20·54 ; 20·70] | 20·21 | [19·93 ; 20·45] |
| Age 18 |  | 21·04 | [20·86 ; 21·20] | 21·20 | [21·13 ; 21·24] | 21·18 | [21·08 ; 21·24] | 20·75 | [20·47 ; 20·99] |
| Age 19 * |  | 21·46 | [21·28 ; 21·62] | 21·62 | [21·55 ; 21·66] | 21·60 | [21·50 ; 21·66] | 21·17 | [20·89 ; 21·41] |
| Age 20 * |  | 21·85 | [21·67 ; 22·01] | 22·01 | [21·94 ; 22·05] | 21·99 | [21·89 ; 22·05] | 21·56 | [21·28 ; 21·80] |

**Table E1.** BMI trajectories for boys after HPSF.

| **HPSF** | ***Control group*** | **Without expert information** | | **With expert information 1** Constant exposure-effect & uncontrolled environment | | **With expert information 2**  Household multiplier & uncontrolled environment  ^π^ | | **With expert information 2**  Household multiplier & household maintainer  ^π^ | |
| --- | --- | --- | --- | --- | --- | --- | --- | --- | --- |
|  | ***Median*** | ***Median*** | ***[LL ; UL]^¥^*** | ***Median*** | ***[LL ; UL]^¥^*** | ***Median*** | ***[LL ; UL]^¥^*** | ***Median*** | ***[LL ; UL]^¥^*** |
| **Girls** ^ǂ^ |  |  |  |  |  |  |  |  |  |
| Age 6 | 15·71 | 15·50 | [15·33 ; 15·66] | 15·51 | [15·39 ; 15·62] | 15·50 | [15·33 ; 15·66] | 15·50 | [15·33 ; 15·66] |
| Age 7 | 16·01 | 15·80 | [15·62 ; 15·95] | 15·81 | [15·69 ; 15·92] | 15·71 | [15·58 ; 15·83] | 15·71 | [15·58 ; 15·83] |
| Age 8 | 16·50 | 16·29 | [16·12 ; 16·45] | 16·30 | [16·19 ; 16·41] | 16·20 | [16·08 ; 16·33] | 16·20 | [16·08 ; 16·33] |
| Age 9 | 16·87 | 16·66 | [16·49 ; 16·82] | 16·67 | [16·56 ; 16·79] | 16·57 | [16·45 ; 16·70] | 16·57 | [16·45 ; 16·70] |
| Age 10 | 17·42 | 17·21 | [17·04 ; 17·37] | 17·22 | [17·11 ; 17·33] | 17·12 | [17·00 ; 17·25] | 17·12 | [17·00 ; 17·25] |
| Age 11 | 18·05 | 17·84 | [17·67 ; 18·00] | 17·85 | [17·74 ; 17·97] | 17·75 | [17·63 ; 17·88] | 17·75 | [17·63 ; 17·88] |
| Age 12 |  | 18·56 | [18·39 ; 18·72] | 18·73 | [18·65 ; 18·77] | 18·71 | [18·61 ; 18·77] | 18·28 | [17·99 ; 18·52] |
| Age 13 |  | 19·17 | [19·00 ; 19·33] | 19·34 | [19·26 ; 19·38] | 19·31 | [19·22 ; 19·37] | 18·88 | [18·60 ; 19·12] |
| Age 14 |  | 19·83 | [19·66 ; 19·99] | 20·00 | [19·92 ; 20·4] | 19·98 | [19·88 ; 20·03] | 19·54 | [19·26 ; 19·78] |
| Age 15 |  | 20·35 | [20·18 ; 20·51] | 20·52 | [20·44 ; 20·56] | 20·50 | [20·40 ; 20·56] | 20·07 | [19·78 ; 20·30] |
| Age 16 |  | 20·87 | [20·70 ; 21·03] | 21·04 | [20·96 ; 21·08] | 21·02 | [20·92 ; 21·08] | 20·59 | [20·30 ; 20·83] |
| Age 17 |  | 21·27 | [21·10 ; 21·43] | 21·44 | [21·36 ; 21·48] | 21·42 | [21·32 ; 21·47] | 20·98 | [20·70 ; 21·22] |
| Age 18 |  | 21·48 | [21·30 ; 21·63] | 21·64 | [21·56 ; 21·68] | 21·62 | [21·52 ; 21·68] | 21·19 | [20·90 ; 21·43] |
| Age 19 * |  | 21·75 | [21·57 ; 21·90] | 21·91 | [21·83 ; 21·95] | 21·89 | [21·79 ; 21·95] | 21·46 | [21·17 ; 21·70] |
| Age 20 * |  | 21·97 | [21·79 ; 22·12] | 22·13 | [22·05 ; 22·17] | 22·11 | [22·01 ; 22·17] | 21·68 | [21·39 ; 21·92] |

**Table E2. BMI trajectories for girls after HPSF.**

**Table E3. BMI trajectories for boys after PAS.**

| **PAS** | ***Control group*** | **Without expert information** | | **With expert information 1** Constant exposure-effect & uncontrolled environment | | **With expert information 2**  Household multiplier & uncontrolled environment  ^π^ | | **With expert information 2**  Household multiplier & household maintainer  ^π^ | |
| --- | --- | --- | --- | --- | --- | --- | --- | --- | --- |
|  | ***Median*** | ***Median*** | ***[LL ; UL] ^¥^*** | ***Median*** | ***[LL ; UL]^¥^*** | ***Median*** | ***[LL ; UL]^¥^*** | ***Median*** | ***[LL ; UL]^¥^*** |
| **Boys** ^ǂ^ |  |  |  |  |  |  |  |  |  |
| Age 6 | 15·45 | 15·28 | [15·12 ; 15·45] | 15·28 | [15·16 ; 15·40] | 15·28 | [15·12 ; 15·45] | 15·28 | [15·12 ; 15·45] |
| Age 7 | 15·30 | 15·13 | [14·97 ; 15·30] | 15·14 | [15·02 ; 15·26] | 15·11 | [15·03 ; 15·19] | 15·11 | [15·03 ; 15·19] |
| Age 8 | 16·89 | 16·72 | [16·56 ; 16·89] | 16·73 | [16·61 ; 16·85] | 16·70 | [16·62 ; 16·78] | 16·70 | [16·62 ; 16·78] |
| Age 9 | 17·14 | 16·97 | [16·81l ; 17·14] | 16·97 | [16·85 ; 17·09] | 16·94 | [16·87 ; 17·02] | 16·94 | [16·87 ; 17·02] |
| Age 10 | 17·61 | 17·44 | [17·28 ; 17·61] | 17·44 | [17·32 ; 17·56] | 17·42 | [17·34 ; 17·49] | 17·42 | [17·34 ; 17·49] |
| Age 11 | 17·94 | 17·78 | [17·61 ; 17·94] | 17·78 | [17·66 ; 17·90] | 17·75 | [17·69 ; 17·83] | 17·75 | [17·69 ; 17·83] |
| Age 12 |  | 18·14 | [17·98 ; 18·31] | 18·27 | [18·20 ; 18·30] | 18·27 | [18·21 ; 18·30] | 18·09 | [17·98 ; 18·19] |
| Age 13 |  | 18·60 | [18·44 ; 18·77] | 18·74 | [18·67 ; 18·77] | 18·73 | [18·67 ; 18·76] | 18·56 | [18·45 ; 18·65] |
| Age 14 |  | 19·08 | [18·92 ; 19·25] | 19·21 | [19·14 ; 19·24] | 19·21 | [19·15 ; 19·24] | 19·03 | [18·92 ; 19·13] |
| Age 15 |  | 19·55 | [19·39 ; 19·72] | 19·68 | [19·61 ; 19·72] | 19·68 | [19·62 ; 19·71] | 19·50 | [19·39 ; 19·60] |
| Age 16 |  | 20·05 | [19·89 ; 20·22] | 20·19 | [20·12 ; 20·22] | 20·18 | [20·12 ; 20·21] | 20·01 | [19·90 ; 20·10] |
| Age 17 |  | 20·54 | [20·37 ; 20·71] | 20·67 | [20·60 ; 20·70] | 20·66 | [20·60 ; 20·70] | 20·49 | [20·38 ; 20·59] |
| Age 18 |  | 21·08 | [20·92 ; 21·25] | 21·21 | [21·14 ; 21·24] | 21·20 | [21·15 ; 21·24] | 21·03 | [20·92 ; 21·13] |
| Age 19 * |  | 21·50 | [21·34 ; 21·67] | 21·63 | [21·56 ;21·66] | 21·62 | [21·57 ; 21·66] | 21·45 | [21·34 ; 21·55] |
| Age 20 * |  | 21·89 | [21·73 ; 22·06] | 22·02 | [21·95 ; 22·05] | 22·01 | [21·96 ; 22·05] | 21·84 | [21·73 ; 21·94] |

| **PAS** | ***Control group*** | **Without expert information** | | **With expert information 1** Constant exposure-effect & uncontrolled environment | | **With expert information 2**  Household multiplier & uncontrolled environment  ^π^ | | **With expert information 2**  Household multiplier & household maintainer  ^π^ | |
| --- | --- | --- | --- | --- | --- | --- | --- | --- | --- |
|  | ***Median*** | ***Median*** | ***[LL ; UL] ^¥^*** | ***Median*** | ***[LL ; UL]^¥^*** | ***Median*** | ***[LL ; UL]^¥^*** | ***Median*** | ***[LL ; UL]^¥^*** |
| **Girls** ^ǂ^ |  |  |  |  |  |  |  |  |  |
| Age 6 | 15·71 | 15·54 | [15·38 ; 15·71] | 15·54 | [15·42 ; 15·66] | 15·51 | [15·44 ; 15·59] | 15·51 | [15·44 ; 15·59] |
| Age 7 | 16·01 | 15·84 | [15·67 ; 16·01] | 15·84 | [15·72 ; 15·96] | 15·81 | [15·73 ; 15·89] | 15·81 | [15·73 ; 15·89] |
| Age 8 | 16·50 | 16·33 | [16·17 ; 16·50] | 16·34 | [16·22 ; 16·46] | 16·31 | [16·23 ; 16·38] | 16·31 | [16·23 ; 16·38] |
| Age 9 | 16·87 | 16·71 | [16·54 ; 16·87] | 16·71 | [16·59 ; 16·83] | 16·68 | [16·60 ; 16·76] | 16·68 | [16·60 ; 16·76] |
| Age 10 | 17·42 | 17·25 | [17·09 ; 17·42] | 17·26 | [17·14 ; 17·38] | 17·23 | [17·15 ; 17·30] | 17·23 | [17·15 ; 17·30] |
| Age 11 | 18·05 | 17·88 | [17·72 ; 18·05] | 17·89 | [17·77 ; 18·01] | 17·86 | [17·78 ; 17·94] | 17·86 | [17·78 ; 17·94] |
| Age 12 |  | 18·61 | [18·44 ; 18·77] | 18·74 | [18·67 ; 18·77] | 18·73 | [18·67 ; 18·77] | 18·56 | [18·45 ; 18·66] |
| Age 13 |  | 19·21 | [19·05 ; 19·38] | 19·34 | [19·27 ; 19·38] | 19·34 | [19·28 ; 19·37] | 19·17 | [19·05 ; 19·26] |
| Age 14 |  | 19·87 | [19·71 ; 20·04] | 20·00 | [19·93 ; 20·04] | 20·00 | [19·94 ; 20·03] | 19·83 | [19·72 ; 19·92] |
| Age 15 |  | 20·40 | [20·23 ; 20·56] | 20·53 | [20·46 ; 20·56] | 21·52 | [20·46 ; 20·56] | 20·35 | [20·24 ; 20·44] |
| Age 16 |  | 20·92 | [20·75 ; 21·08] | 21·05 | [20·98 ; 21·08] | 21·04 | [20·98 ; 21·08] | 20·87 | [20·76 ; 20·97] |
| Age 17 |  | 21·31 | [21·15 ; 21·48] | 21·44 | [21·37 ; 21·48] | 21·44 | [21·38 ; 21·47] | 21·27 | [21·16 ; 21·36] |
| Age 18 |  | 21·52 | [21·35 ; 21·69] | 21·65 | [21·58 ; 21·68] | 21·64 | [21·58 ; 21·68] | 21·47 | [21·36 ; 21·57] |
| Age 19 * |  | 21·79 | [21·62 ; 21·96] | 21·92 | [21·85 ; 21·95] | 21·94 | [21·85 ; 21·95] | 21·74 | [21·63 ; 21·84] |
| Age 20 * |  | 22·01 | [21·84 ; 22·18] | 22·14 | [22·07 ; 22·17] | 22·13 | [22·07 ; 22·17] | 21·96 | [21·85 ; 22·06] |

**Table E4. BMI trajectories for girls after PAS.**

**Notes:** BMI = body mass index, CI = confidence interval, FDGS = Fifth Dutch Growth Study, HPSF = Healthy Primary School of the Future, IOTF = International Obesity Task Force, LL = lower bound (based on lower bound of effect size), PAS = Physical Activity School, UL = upper bound (based on upper bound of effect size), SES = socioeconomic status.
* Calibration based on the trend in the Fifth Dutch Growth Study (see main text).
***^¥^*** based on lower limit and upper limit of the BMI reduction corresponding to the uncertainty scenarios.
^ǂ^ Boys, model 2; Girls, model 1.
^Π^ Household multiplier applied after the third year of intervention implementation (age 7 years).

Appendix F**. Details on effect maintenance scenarios: a selection of relevant literature**

| **Period:** Primary school period, 4-12 years of age **Uncertainty:** uncertainty on the (unobserved) relative effects while exposed | |
| --- | --- |
| **Scenario** | **A selection of relevant literature** |
| 1. Constant exposure- effect | Description: The evidence base on the maintenance of intervention effects is (relatively) poor. It is most likely that the intervention effects are expected to stay about the same during the entire primary school-period if exposure is prolonged. First of all, children adopt a new ‘social norm’ more easily as compared to adults.^10^ After a 2-year period, children are probably used to the adopted behaviour changes. When exposure to the new ‘social norm’ is continued, it is likely that the effects will stay about the same. Body weight changes resulting from reductions in the energy imbalance (e.g. reduced energy intake or increased energy expenditure) generally reach a ‘plateau’ level after about 2 years.^11^ In addition to the school environment, health behaviours of parents and peers are important determinants of children’s health behaviours (environment).^12,13^ If family practices stay the same, and if most of the closest peers come from the same schools, it is likely that the relative effects (intervention vs. control) remain stable. |
| 1. Household multiplier | Continued intervention exposure may not only lead to effect maintenance, but may also induce health behaviour, predominantly via transfer of behaviours to the household setting. Spillover effects may occur, as obesity and related lifestyle behaviours spread through social networks.^14^ With continued exposure, children and parents may get more used to the changed health behaviours, and it may become easier for them to adopt them outside of the school setting. Spillover effects may be more likely for interventions that aim at changing the whole environment/system, like whole-school approaches, as compared to educational interventions, because whole-school approaches focus on making multiple changes (physical environment, school policy, community involvement, parental involvement etc.) to induce a ‘cultural shift’ among children, teachers, and parents. The transfer of behavioural changes is also influenced by environmental circumstances (e.g. beliefs, knowledge, habits in the household). |
| 1. *Personal factors* | In addition to getting used to new lifestyle behaviours, the interventions might have an impact on other internal/psychosocial mechanisms (e.g. self-regulation, ‘not feeling comfortable’ with participating in sports). In addition, interventions might influence children’s motoric abilities, and fitness levels. These impacts can potentially contribute to some sustained effects on behaviour changes and BMI z-scores. |
| **Period:** After the primary school period, 12 up to 20 years of age **Uncertainty:** Uncertainty on the (unobserved) relative effect after intervention exposure | |
| 1. Uncontrolled environment | The effect maintenance after the primary school period is uncertain. After the primary school period, children will be exposed to an uncontrolled environment, which affects their lifestyle behaviours. Important factors in this environment such as behaviours of peers and parents (e.g. family routines, peer pressure), circumstances and physical characteristics at school (e.g. foods provided at school, proximity to supermarkets) and at home (e.g. availability of foods) may affect the effect maintenance.^15-21^  In this ‘obesogenic environment’, it is difficult to maintain behaviour changes. In secondary school, sport participation rates decline, and dietary behaviours become unhealthier.^22,23^ In addition, children become more autonomous, and peer influences of (non-exposed) peers will increase.^13^ All together, the relative intervention effects between the intervention and control group will probably decrease. The effects will decay within the first year of secondary school, and will then reach a plateau level. Potentially, children may adopt the behaviours changes again in young adulthood when starting a family life or career.^24^ At this time point, individual circumstances change which lead to deliberately considering health behaviours after a period of habit discontinuity.^25,26^  The evidence base on long-term effect maintenance is poor, and the extent of effect maintenance is hard to predict, because of the multifactorial aspects during the teenage and adolescence period. |
| 1. Household maintainer | Long-term exposure to health behaviours in the school setting can influence the household setting in which children grow up (see household multiplier scenario). When effects transfer to the household, it is also likely that they will be (somewhat) maintained. The effect transfer particularly pertains to continued interventions with a combination of nutrition and diet and extensive parental involvement, as transfer of dietary behaviours is most likely. The transfer of health behaviours to the household may be disproportionally distributed across family (socioeconomic) backgrounds with potentially disadvantaging low socioeconomic status groups.^17^ |

**References**

1. Knol AB, Slottje P, van der Sluijs JP, Lebret E. The use of expert elicitation in environmental health impact assessment: a seven step procedure. Env Health 2010; 9: 19.
2. Tong A, Sainsbury P, Craig J. Consolidated criteria for reporting qualitative research (COREQ): a 32-item checklist for interviews and focus groups. Int J Qual Health Care 2007; 19: 349–57.
3. Cooke R, Probst KN. Highlights of the Expert Judgment Policy Symposium and Technical Workshop. Conference summary; 2006. https://media.rff.org/documents/Conference-Summary.pdf Date accessed: March 01, 2020.
4. Morris DE, Oakley JE, Crowe JA. A web-based tool for eliciting probability distributions from experts. Env Model Sof 2014; 52: 1–4.
5. Pawson R, Tilley N. Realist Evaluation. Sage Publications Ltd. 2004. http://www.communitymatters.com.au/RE_chapter.pdf Date accessed: March 01, 2020.
6. Birt L, Scott S, Cavers D, Campbell C, Walter F. Member Checking: A Tool to Enhance Trustworthiness or Merely a Nod to Validation? Qual Health Res 2016; 26: 1802–11.
7. Slottje P, Van der Sluijs JP, Knol AB. Expert Elicitation. Methodological suggestions for its use in environmental health impact assessments. Bilthoven: National Institute for Public Health and the Environment. 2008. https://www.rivm.nl/bibliotheek/rapporten/630004001.html. Accessed February 10, 2020.
8. Usher W, Strachan N. An expert elicitation of climate, energy and economic uncertainties. Energy Policy 2013; 61: 811–21.
9. Cole TJ, Bellizzi MC, Flegal KM, Dietz WH. Establishing a standard definition for child overweight and obesity worldwide: international survey. BMJ 2000; 320:1240–3.
10. Rose G. Sick individuals and sick populations. Int J Epid 2001; 30: 427–32.
11. de Ruyter JC, Olthof MR, Seidell JC, Katan MB. A trial of sugar-free or sugar-sweetened beverages and body weight in children. New Engl J Med 2012; 367: 1397–406.
12. de Heer HD, Koehly L, Pederson R, Morera O. Effectiveness and spillover of an after-school health promotion program for Hispanic elementary school children. Am J Pub Health 2011; 101: 1907–13.
13. Mollborn S, Lawrence E. Family, Peer, and School Influences on Children's Developing Health Lifestyles. J Health Soc Behav.2018; 59: 133–50.
14. Christakis NA, Fowler JH. The spread of obesity in a large social network over 32 years. New Eng J Med 2007; 357: 370–9.
15. Brown HE, Corder K, Atkin AJ, van Sluijs EMF. Childhood predictors of adolescent behaviour: The prospective association of familial factors with meeting physical activity guidelines. Prev Med Rep 2017; 6: 221–7.
16. Ovrebo B, Stea TH, Te Velde SJ, Bjelland M, Klepp KI, Bere E. A comprehensive multicomponent school-based educational intervention did not affect fruit and vegetable intake at the 14-year follow-up. Prev Med 2019; 121: 79–85.
17. Plachta-Danielzik S, Landsberg B, Lange D, Seiberl J, Muller MJ. Eight-year follow-up of school-based intervention on childhood overweight--the Kiel Obesity Prevention Study. Obes Facts 2011; 4: 35–43.
18. Taylor RW, McAuley KA, Barbezat W, Farmer VL, Williams SM, Mann JI. Two-year follow-up of an obesity prevention initiative in children: the APPLE project. Am J Clin Nutr 2008; 88: 1371–7.
19. Kafatos A, Manios Y, Moschandreas J. Health and nutrition education in primary schools of Crete: follow-up changes in body mass index and overweight status. Eorp J Clin Nutr 2005; 59: 1090–2.
20. Neumark-Sztainer D, Story M, Hannan PJ, Rex J. New Moves: a school-based obesity prevention program for adolescent girls. Prev Med 2003; 37: 41–51.
21. James J, Thomas P, Kerr D. Preventing childhood obesity: two year follow-up results from the Christchurch obesity prevention programme in schools (CHOPPS). Bmj 2007; 335: 762.
22. Nader PR, Bradley RH, Houts RM, McRitchie SL, O'Brien M. Moderate-to-vigorous physical activity from ages 9 to 15 years. Jama 2008; 300: 295–305.
23. Winpenny EM, van Sluijs EMF, White M, Klepp K-I, Wold B, Lien N. Changes in diet through adolescence and early adulthood: longitudinal trajectories and association with key life transitions. Int J Behav Phys Act 2018; 15:86.
24. Stea TH, Tveter ET, Te Velde SJ, Vik FN, Klepp KI, Bere E. The effect of an extra piece of fruit or vegetables at school on weight status in two generations - 14 years follow-up of the Fruit and Vegetables Makes the Marks study. PLoS One 2018; 13: e0205498.
25. Verplanken B, Walker I, Davis A, Jurasek M. Context change and travel mode choice: Combining the habit discontinuity and self-activation hypotheses. J Env Psych 2008; 28: 121–7.
26. Marcus BH, Dubbert PM, Forsyth LH, et al. Physical activity behavior change: issues in adoption and maintenance. Health Psychol 2000; 19: 32–41.
